# Degree of regional variation and effects of health insurance-related factors on the utilization of 24 diverse healthcare services - a cross-sectional study

**DOI:** 10.1186/s12913-020-05930-y

**Published:** 2020-11-27

**Authors:** Wenjia Wei, Agne Ulyte, Oliver Gruebner, Viktor von Wyl, Holger Dressel, Beat Brüngger, Eva Blozik, Caroline Bähler, Julia Braun, Matthias Schwenkglenks

**Affiliations:** 1 Department of Epidemiology, Epidemiology, Biostatistics & Prevention Institute, University of Zurich, Hirschengraben 84, 8001 Zurich, Switzerland; 2 Department of Geography, University of Zurich, Winterthurerstrasse 190, 8057 Zurich, Switzerland; 3 Division of Occupational and Environmental Medicine, Department of Epidemiology, Epidemiology, Biostatistics & Prevention Institute, University of Zurich and University Hospital Zurich, Hirschengraben 84, 8001 Zurich, Switzerland; 4 Department of Health Sciences, Helsana Group, Zürichstrasse 130, 8600 Dübendorf, Switzerland; 5 Institute of Primary Care, University of Zurich and University Hospital Zurich, Pestalozzistrasse 24, 8091 Zürich, Switzerland; 6 Departments of Epidemiology and Biostatistics, Epidemiology, Biostatistics & Prevention Institute, University of Zurich, Hirschengraben 84, 8001 Zurich, Switzerland

**Keywords:** Regional variation, Healthcare utilization, Influencing factors, Health insurance

## Abstract

**Background:**

Regional variation in healthcare utilization could reflect unequal access to care, which may lead to detrimental consequences to quality of care and costs. The aims of this study were to a) describe the degree of regional variation in utilization of 24 diverse healthcare services in eligible populations in Switzerland, and b) identify potential drivers, especially health insurance-related factors, and explore the consistency of their effects across the services.

**Methods:**

We conducted a cross-sectional study using health insurance claims data for the year of 2014. The studied 24 healthcare services were predominantly outpatient services, ranging from screening to secondary prevention. For each service, a target population was identified based on applicable clinical recommendations, and outcome variable was the use of the service. Possible influencing factors included patients’ socio-demographics, health insurance-related and clinical characteristics. For each service, we performed a comprehensive methodological approach including small area variation analysis, spatial autocorrelation analysis, and multilevel multivariable modelling using 106 mobilité spaciale regions as the higher level. We further calculated the median odds ratio in model residuals to assess the unexplained regional variation.

**Results:**

Unadjusted utilization rates varied considerably across the 24 healthcare services, ranging from 3.5% (osteoporosis screening) to 76.1% (recommended thyroid disease screening sequence). The effects of health insurance-related characteristics were mostly consistent. A higher annual deductible level was mostly associated with lower utilization. Supplementary insurance, supplementary hospital insurance and having chosen a managed care model were associated with higher utilization of most services. Managed care models showed a tendency towards more recommended care. After adjusting for multiple influencing factors, the unexplained regional variation was generally small across the 24 services, with all MORs below 1.5.

**Conclusions:**

The observed utilization rates seemed suboptimal for many of the selected services. For all of them, the unexplained regional variation was relatively small. Our findings confirmed the importance and consistency of effects of health insurance-related factors, indicating that healthcare utilization might be further optimized through adjustment of insurance scheme designs. Our comprehensive approach aids in the identification of regional variation and influencing factors of healthcare services use in Switzerland as well as comparable settings worldwide.

**Supplementary Information:**

The online version contains supplementary material available at 10.1186/s12913-020-05930-y.

## Background

A recent systematic review found a substantial evidence base for large variation in the utilization of healthcare services across regions, hospitals, and healthcare providers [1]. The ubiquity and persistence of such variation cannot simply be explained by variation in the actual care needs of different populations [2]. A substantial portion may reflect inappropriate variability due to unequal access to care, potentially detrimental for quality of care and costs [3]. This unwarranted component, although difficult to quantify, should be minimized in order to improve the quality, equity, and efficiency of healthcare [4]. Regional variation may be driven by multiple factors, including patient socio-demographics, clinical characteristics, availability of physicians and healthcare facilities, and healthcare system-related factors [2]. They can function as personal, financial, and organizational modifiers of access to care [5]. System-related factors (e.g. relating to health insurance systems, national legislation or programs) are of strong interest, because their modification may offer big levers to reduce unwarranted variation at a national level. We would regard related findings as more meaningful and instructive where same direction of effects is observed across diverse healthcare services.

Existing studies of regional variation in utilization mostly applied methods of small area variation analysis (SAVA) [1]. Several problems were identified. First, numerous analyses focused on only part of a country, without nationwide coverage. Second, the selection of studied healthcare services was often arbitrary and opportunity-driven, suggesting that future studies should focus on services of high clinical importance, policy relevance, and public awareness. Third, the causes and drivers of variation were rarely explored. Only few studies controlled for a limited number of possible influencing factors such as individual socio-demographics or clinical characteristics [6, 7]. Finally, most studies assessed the variation in utilization of a single service, or one category of services (e.g. related surgical procedures) [8, 9]. More comprehensive studies simultaneously comparing a wider range of services are currently missing. Around 40% of studies of variation in healthcare utilization used administrative data routinely collected for billing purposes. Although subject to certain limitations (e.g. restricted clinical information), health insurance claims data play an important role in health services research [10, 11].

In the present study, we aimed to select a variety of healthcare services and their target populations based on applicable clinical recommendations, and to describe the degree of regional variation in their utilization in Switzerland, a system with universal care access and high out-of-pocket expenditures, using claims data. Specific goals were to a) evaluate the degree of unadjusted and adjusted regional variation in utilization in eligible populations, b) identify potential influencing factors, especially health insurance-related characteristics, and c) explore the consistency of these factors’ effects across the selected services.

## Methods

### Selection of healthcare services and eligible populations

Our study focused on primary healthcare for major non-communicable diseases, and the selection of healthcare services and the identification and extraction of the corresponding eligible populations were based on a systematic approach described earlier [12]. Recommendation statements from clinical practice guidelines of Swiss, European and relevant international medical societies, used in Switzerland, were considered pragmatically according to clinical relevance, expected frequency of service use, size of the eligible population, and feasibility to identify the population and service from Swiss health insurance claims data. Some services outside primary healthcare were included to extend the spectrum of populations investigated and reflect services currently debated in Switzerland.

The final selection consisted of 24 services reflecting different categories of care, including screening (*N* = 4), diagnosis (*N* = 6), primary prevention (*N* = 1), treatment (*N* = 4) and secondary prevention (*N* = 9). Table 1 lists descriptions of services, eligible populations, and recommendation status, with details relevant for the extraction of eligible study populations and service utilization information from Swiss health insurance claims data through a systematic approach.

**Table 1:** Definition and description of selected 24 healthcare services

Category	Healthcare service	Service description and frequency	Study population	Recommendation
Screening	Colon cancer screening	Colonoscopy/ year	Anyone 50–69 years old	Colonoscopy should be done every 10 years for people 50–69 years old.
	Breast cancer screening	Mammography/ year	50–74 years old women	Mammography should be done every 2 years for 50–74 years old women.
	Prostate cancer screening	Prostate-specific antigen (PSA) testing/ year	50–70 years old men	Routine prostate cancer screening with PSA testing is not recommended.
	Osteoporosis screening	Dual-energy x-ray absorptiometry (DXA)/ year	People over 60 and with risk factors^a^ of spontaneous fractures	DXA densitometry is recommended for patients with spontaneous fractures or increased risk of them.
Diagnosis	DM: HbA1c test	Glycated hemoglobin (HbA1c) test twice/ year	Adult drug-treated diabetes patients	HbA1c test should be done for diabetes patients at least twice a year.
	DM: kidney exam	Albuminuria and serum creatinine tests/ year	Adult drug-treated diabetes patients	Albuminuria and serum creatinine tests should be done for diabetes patients at least once a year.
	DM: LDL test	Low-density lipoprotein (LDL) test/ year	Adult drug-treated diabetes patients under 75 years old	LDL test should be done for diabetes patients at least once a year.
	DM: eye check	Ophthalmologist visit/ year	Adult drug-treated diabetes patients	Eye exam should be performed for diabetes patients at least once a year.
	TSH	Thyroid stimulating hormone (TSH) test without T3 and T4 tests on the same day	Adults without thyroid disease and receiving TSH test	TSH should be measured as an initial screening test for hypo/hyperthyroidism, while T3 and T4 test should follow if TSH is abnormal.
	POCR	Outpatient preoperative chest radiography (POCR) up to 2 months before surgery	Adult patients with inpatient surgical procedures	Routine chest radiography is not recommended before surgery.
Primary prevention	Influenza vaccination	Influenza outpatient vaccination/ year	People over 65 years old or with a specified chronic condition^b^	People over 65 years old and patients with chronic conditions, specified by Federal Office of Public Health, should be vaccinated against influenza every year.
Treatment	BZD	Cumulative prescription of benzodiazepines (BZD) for > 8 weeks/ year	Anyone over 65 years old	Long-term use of benzodiazepines and other hypnotics is discouraged for old patients.
	PPI	Cumulative prescription of proton pump inhibitors (PPI) or H2 histamine receptor antagonists (H2) for > 8 weeks/ year	Adults receiving PPI or H2 drugs	PPI should not be used at maximal dose for prolonged periods of time.
	Outpatient procedures	Specified surgical procedures^c^ done in the outpatient setting	Adult patients with specified surgical procedures (either as in- or outpatient)	If none of the special conditions apply, certain surgical procedures should be done in the outpatient setting.
	C-section	Cesarean section (C-section)	Women giving birth without absolute indications^d^ for C-section	C-section should not be performed unless absolute or relative indications are present.
Secondary prevention	AMI: aspirin	Aspirin prescription within 2 weeks after acute myocardial infarction (AMI)	Adult patients with AMI	All myocardial infarction patients should take aspirin long-term.
	AMI: statin	High-dose statin prescription within 2 weeks after AMI	Adult patients with AMI	All myocardial infarction patients should get statins long-term.
	AMI: beta-blocker	Beta-blocker prescription within 2 weeks after AMI	Adult patients with AMI	All myocardial infarction patients with heart failure or impaired function should get beta-blockers long-term.
	AMI: ACE/ARB	Angiotensin converting enzyme (ACE) or angiotensin receptor blocker (ARB) antihypertensive medication prescription within 2 weeks after AMI	Adult patients with AMI	All myocardial infarction patients with heart failure or impaired function should get ACE or ARB antihypertensive medication long-term.
	AMI: P2Y	P2Y antiplatelet drug^e^ prescription within 2 weeks after AMI	Adult patients with AMI	All myocardial infarction patients should get P2Y antiplatelet drugs for at least 1–12 months according to the bleeding risk profile and AMI treatment.
	PPI with NSAID	PPI prescription within 1 month or up to 3 months before initial long-term nonsteroidal anti-inflammatory drug (NSAID) prescription	Adult patients with a cumulative NSAID prescription of > 8 weeks at maximal dose	Patients taking long-term NSAID and with risk factors for gastric ulcer^f^ should also take PPI.
	PAD: statin	Prescription of statins within 3 months after peripheral artery disease (PAD) identification	Adult patients undergoing diagnostic or treatment procedures for PAD	Statins are recommended for all patients with PAD.
	Afib: anticoagulation	Oral anticoagulation prescription within 2 weeks after atrial fibrillation (Afib) identification	Adult patients with atrial fibrillation diagnosis and additional risk factors^g^	All patients with atrial fibrillation should be prescribed oral anticoagulation for embolic events prevention according to the CHA_2_DS_2_-VASc score.
	GKK	Glucocorticoid (GKK) prescription within 1 month or up to 3 months before disease-modifying antirheumatic drug (DMARD) prescription	Adult patients with a new prescription of DMARD by a rheumatologist	Short-term glucocorticoids should be taken with newly prescribed DMARD.

^a^. Recent distal radius, proximal humerus, vertebral or femoral fracture, use of drugs increasing the risk of osteoporosis, use of oral glucocorticoids, diabetes, ankylosing spondylitis, osteogenesis imperfecta, rheumatoid arthritis, inflammatory bowel disease, Cushing’s disease, alcohol or nicotine abuse, chronic liver disease, gastrectomy, malnutrition, hypogonadism, hyper- or hypothyroidism, and hyperparathyroidism. Patients currently treated or diagnosed with osteoporosis were excluded

^b^. Cardiovascular disease, chronic pulmonary disease, diabetes, chronic liver disease, renal failure, immune deficiency, systemic neurologic disorders

^c^. Varicose veins ligation and stripping, surgical procedures of hemorrhoids, inguinal hernia and cervix, knee arthroscopy and meniscectomy, tonsillectomy

^d^. Placental, umbilical cord or fetal pathology, HIV or genital HSV infection, or multiple pregnancy

^e^. Clopidogrel, prasugrel or ticagrelor

^f^. Concurrent use of antiplatelet, anticoagulant drugs, oral glucocorticoids or recent hospitalization with any major bleeding

^g^. Risk factors (congestive heart failure, hypertension, age 65–74 or ≥ 75 years old, diabetes, previous stroke, transient ischemic attack, or thromboembolism, cardiovascular disease, female sex) were extracted from available claims data and summed according to CHA_2_DS_2_-VASc score. Patients with CHA_2_DS_2_-VASc score of ≥2 for males and ≥ 3 for females were included

*DM* Diabetes mellitus

### Study design and populations

Our cross-sectional study used mandatory health insurance claims data provided by Helsana, one of the largest health insurers in Switzerland. The underlying database covered around 1.2 million people, 15% of the Swiss population. The eligible population for each healthcare service was identified from persons enrolled with Helsana during 2014 (Table 1). Asylum seekers, Helsana employees, enrolees living outside Switzerland, with incomplete address information, or living in nursing homes with lump-sum reimbursement of some healthcare services were excluded.

Swiss mandatory health insurance covers a federally defined, uniform benefit package for anyone living in Switzerland regardless of health status. A higher annual deductible (of Swiss Francs 500, 1000, 1500, or 2500) can be chosen instead of the legal minimum of 300, implying lower premiums. People can also choose between standard fee-for-service and managed care models [13, 14], the latter requiring a specific general practitioner or telemedicine provider as the first contact for a new health problem, and resulting in lower premiums. In addition to mandatory insurance, a variety of supplementary insurance products can be bought, for instance, supplementary hospital insurance allowing for hospitalization in semiprivate or private wards.

The data provided by Helsana were anonymized. According to the national ethical and legal regulations, ethical approval was not needed for this type of analysis. This was confirmed by a waiver of the competent ethics committee (Kantonale Ethikkommission Zürich, dated 11th January 2017, BASEC-Nr. Req-2017-00011).

### Outcome and explanatory variables

For each of the selected services, the outcome variable was whether the service was used by each member of the eligible population (Table 1). Candidate explanatory variables available for all 24 healthcare services included a) socio-demographics, i.e. age, gender, language region, purchasing power index, and urban/rural residence, b) health insurance-related characteristics, including having any supplementary insurance, having supplementary hospital insurance, choice of a standard or managed care model, choice of annual deductible, c) number of chronic comorbidities as indicated by pharmaceutical cost groups [15]. In people with supplementary hospital insurance, we could not distinguish the additional presence of other supplementary insurances but only evaluate a mixed effect. To verify the effect of supplementary hospital insurance, we performed sensitivity analyses using different combinations of available explanatory variables. We further included service-specific clinical conditions of relevance and a few service-specific non-individual level variables. We could include regional-level factors in the analysis for four selected healthcare services. For preoperative chest radiography (POCR), the type of hospital performing the surgery (central, primary, surgical, or other specialized hospital) was considered. For breast cancer screening, we determined if a cantonal-level breast cancer screening program existed. Analysis of eye examinations in diabetes patients considered ophthalmologist density per 10,000 inhabitants in each region. For surgical procedures recommended to be performed in outpatient settings, hospital bed density per 1000 inhabitants in each region was considered.

### Geographic units

We used spatial mobility regions (mobilité spatiale - MS) as the geographic level of analysis (*N* = 106). MS regions are defined by the Swiss Federal Statistical Office and used as intermediate-size units of analysis for scientific and regional policy purposes [16]. Each study participant’s residence was assigned to the corresponding MS region.

### Statistical analysis

A four-step analytical approach was applied to all selected healthcare services. In the first step, we descriptively analysed each study population’s characteristics.

Second, we calculated raw utilization rates per MS region and described the degree of regional variation using small area variation analysis (SAVA). We computed extremal quotient (EQ), interquartile range (IQR), coefficient of variation (CV) and systematic component of variation (SCV). SCV estimates the systematic component of variation between small regions by subtracting the component of random variation from total variation, considering age and sex [8, 17]. SCV has been demonstrated to perform generally well in the identification and quantification of variation beyond chance, and has often been used to compare the variability of different healthcare services [18, 19]. Compared to EQ, IQR and CV, which are simple descriptive statistics, SCV is considered more reliable. Therefore, we used SCV as the main estimate to evaluate the degree of regional variation in healthcare utilization before multivariable adjustment. SCV values above 3, between 5.4 and 10, and above 10 suggest relevant, considerable, and very high variation in utilization, respectively [20]. We further checked spatial autocorrelation of regional utilization rates with global Moran’s I statistic [21]. Moran’s I measures the correlation of a variable with itself across space, ranging from − 1 to 1. Moran’s I close to 0 suggests random distribution across space. Significantly positive (negative) Moran’s I values indicate that neighbouring regions are more similar (dissimilar) than distant regions.

Third, to correctly represent the nested data structure, to have more precise standard error estimates, and to assess the variation between regional units, we performed two-level logistic regression modelling with individuals as the lower-level and MS regions as the higher-level of analysis. For POCR, a cross-classified model was developed, with hospitals where surgeries were performed as an additional level cross-classified with MS regions, as we assumed an impact of hospitals on POCR utilization [22]. A sensitivity analysis used our standard, two-level approach. Inclusion of explanatory variables was based on the deviance information criterion [23]. We calculated multivariable-adjusted odds ratios (ORs) and 95% confidence intervals (95%CIs) to estimate the effect of explanatory variables on utilization.

In the last step, we assessed the degree of unexplained regional variation after multilevel modelling by calculating median odds ratios (MORs) and variance partition coefficients (VPCs). MOR is extrapolated from the variance of random effects in multilevel models. It compares the adjusted odds of using the analysed service in two individuals with identical characteristics, but living in two randomly selected regions. The median of all possible resulting ORs is defined as MOR. MOR is always above one, as the higher-propensity region is always compared with the lower-propensity region for the outcome of interest. VPC represents the proportion of total variation accounted for systematic differences between MS regions. The interpretation of the magnitude of MOR should be related to VPC [24]. A relatively big MOR in combination with a considerable VPC indicates substantial regional variation [24]. In addition, we checked for spatial autocorrelation in model residuals across MS regions to check the modelling assumption of independence of regional units, again using global Moran’s I statistic [25, 26].

Statistical analyses were performed using R 3.4.4 [27], STATA 13, and MLwiN 3.04 [28] integrated in STATA using the runmlwin package [29]. Spatial autocorrelation analysis was done with GeoDa 1.10 [30]. The results from all selected healthcare services were finally compared graphically.

## Results

### Study populations

Across the 24 selected healthcare services, eligible population size ranged from 409,960 for influenza vaccination to 1992 for new prescription of a disease-modifying anti-rheumatic drug (DMARD) that should be prescribed concomitantly with a glucocorticoid (Table 2). The mean age of populations ranged from 31.9 years (women giving birth without absolute indications for C-section), to 80.8 years (patients with atrial fibrillation and indication for oral anticoagulation). Overall utilization varied from 3.5% of older people with risk factors for fractures receiving osteoporosis screening, to 76.1% of eligible people receiving a thyroid-stimulating hormone (TSH) test as recommended.

**Table 2:** Basic characteristics of study populations for selected 24 healthcare services

Service category	Healthcare service	Total Number	Age (mean, sd)	Female gender	Utilization rate
Screening	Colon cancer screening	276,387	58.6 (5.8)	142,675 (51.6%)	5.9%
	Breast cancer screening	178,145	61.0 (7.2)	–	20.9%
	Prostate cancer screening	145,874	59.1 (6.2)	–	28.4%
	Osteoporosis screening	97,237	72.5 (8.5)	60,812 (62.5%)	3.5%
Diagnosis	DM: HbA1c test	49,198	66.6 (13.0)	22,138 (45.0%)	69.6%
	DM: kidney exam	49,198	66.6 (13.0)	22,138 (45.0%)	44.3%
	DM: LDL test	33,975	60.1 (11.2)	13,977 (41.2%)	44.3%
	DM: eye check	49,198	66.6 (13.0)	22,138 (45.0%)	55.5%
	TSH	169,232	56.8 (18.5)	111,847 (66.1%)	76.1%
	POCR	47,215	60.3 (17.2)	27,086 (57.4%)	13.0%
Primary prevention	Influenza vaccination	409,960	64.1 (16.3)	230,202 (56.2%)	20.9%
Treatment	BZD	243,951	75.0 (7.6)	141,986 (58.2%)	18.6%
	PPI	153,523	55.7 (17.8)	93,543 (60.9%)	55.5%
	Outpatient procedures	10,656	50.5 (13.7)	7719 (72.4%)	61.4%
	C-section	9449	31.9 (5.1)	–	28.5%
Secondary prevention	AMI: aspirin	2232	72.4 (13.7)	801 (35.9%)	47.0%
	AMI: statin	2232	72.4 (13.7)	801 (35.9%)	34.2%
	AMI: beta-blocker	2232	72.4 (13.7)	801 (35.9%)	42.1%
	AMI: ACE/ARB	2232	72.4 (13.7)	801 (35.9%)	43.8%
	AMI: P2Y	2232	72.4 (13.7)	801 (35.9%)	46.8%
	PPI with NSAID	95,072	61.0 (16.2)	60,804 (64.0%)	43.5%
	PAD: statin	23,868	63.6 (16.5)	12,113 (50.7%)	28.5%
	Afib: anticoagulation	8291	80.8 (7.9)	4037 (48.7%)	27.5%
	GKK	1992	59.2 (15.3)	1369 (68.7%)	58.7%

*sd* Standard deviation, *DM* Diabetes mellitus, *HbA1c* Glycated hemoglobin, *LDL* Low-density lipoprotein, *TSH* Thyroid stimulating hormone, *POCR* Outpatient preoperative chest radiography, *BZD* Benzodiazepines, *PPI* Proton pump inhibitor, *C-section* Cesarean section, *AMI* Acute myocardial infarction, *ACE* Angiotensin converting enzyme, *ARB* Angiotensin receptor blocker, *P2Y* Clopidogrel, prasugrel or ticagrelor, *NSAID* Nonsteroidal anti-inflammatory drug, *PAD* Peripheral artery disease, *Afib* Atrial fibrillation, *GKK* Glucocorticoid

### Effects of explanatory variables

After multivariable adjustment, we observed inconsistent associations between socio-demographic variables and healthcare utilization (Additional file 1: Supplementary Table 1). Age showed a nonlinear effect in most cases; utilization typically reached a peak between 50 and 70 years and then decreased (Additional file 3: Supplementary Fig. 1). Gender effects were mostly not prominent. However, there was an OR of 3.66 (95%CI: 3.10, 3.99) for osteoporosis screening use in women, while the OR for statin prescription in women with peripheral artery disease was 0.52 (95%CI: 0.49, 0.55). Having more comorbidities was significantly associated with increased use of most services, but not secondary prevention medication after acute myocardial infarction (AMI) or oral anticoagulation in patients with atrial fibrillation.

**Fig. 1:**
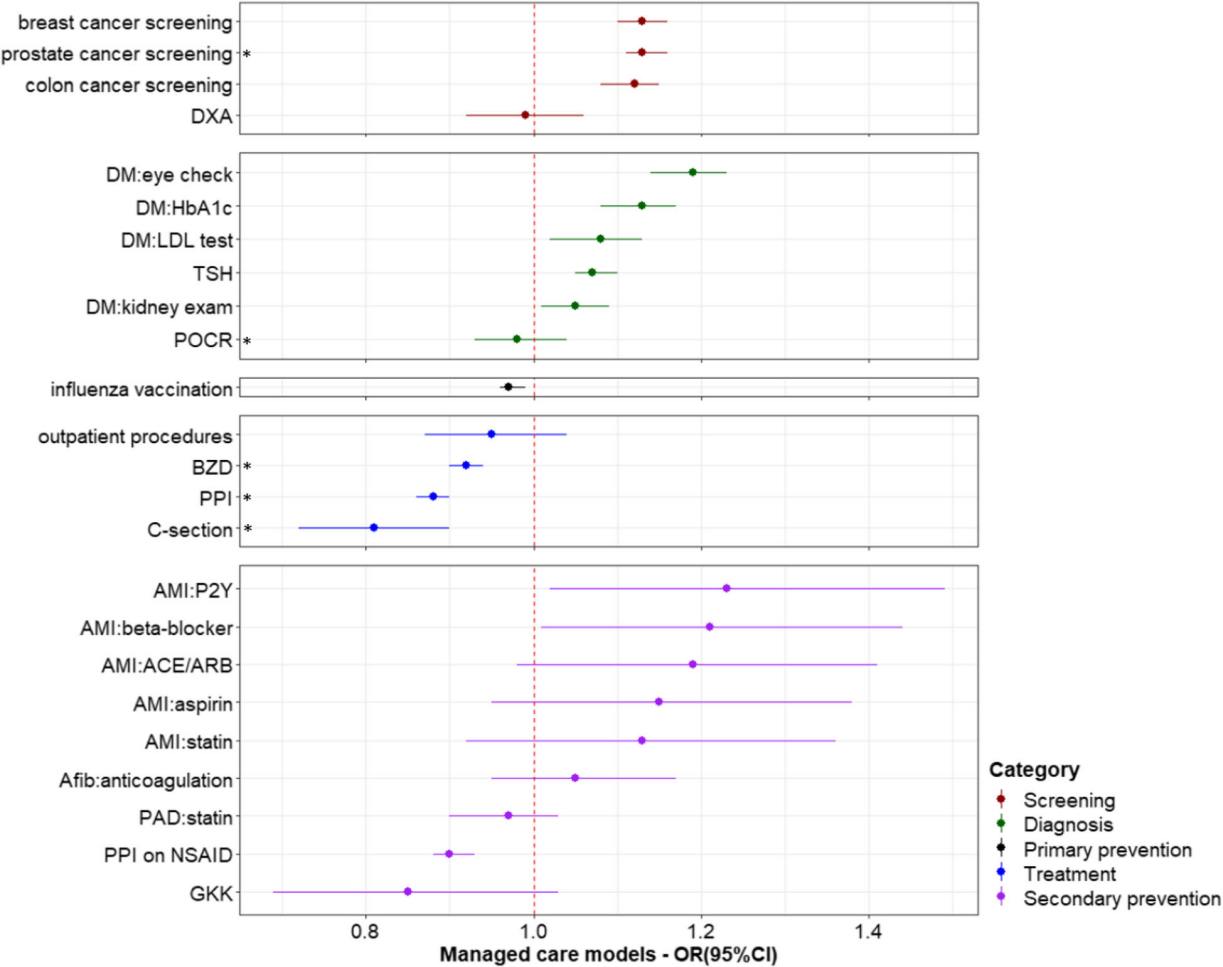
Effects of managed care models on healthcare services utilization. *Indicates services that are discouraged and therefore an odds ratio < 1 indicates better conformity with recommendations, for all other services, an odds ratio > 1 indicates greater use and better guideline conformity. OR: odds ratio; CI: confidence interval; DM: diabetes mellitus; DXA: Dual-energy x-ray absorptiometry; HbA1c: glycated hemoglobin; LDL: low-density lipoprotein; TSH: thyroid stimulating hormone; POCR: outpatient preoperative chest radiography; BZD: benzodiazepines; PPI: proton pump inhibitor; C-section: Cesarean section; AMI: acute myocardial infarction; ACE: angiotensin converting enzyme; ARB: angiotensin receptor blocker; P2Y: clopidogrel, prasugrel or ticagrelor; NSAID: nonsteroidal anti-inflammatory drug; PAD: peripheral artery disease; Afib: atrial fibrillation; GKK: Glucocorticoid

**Fig. 2:**
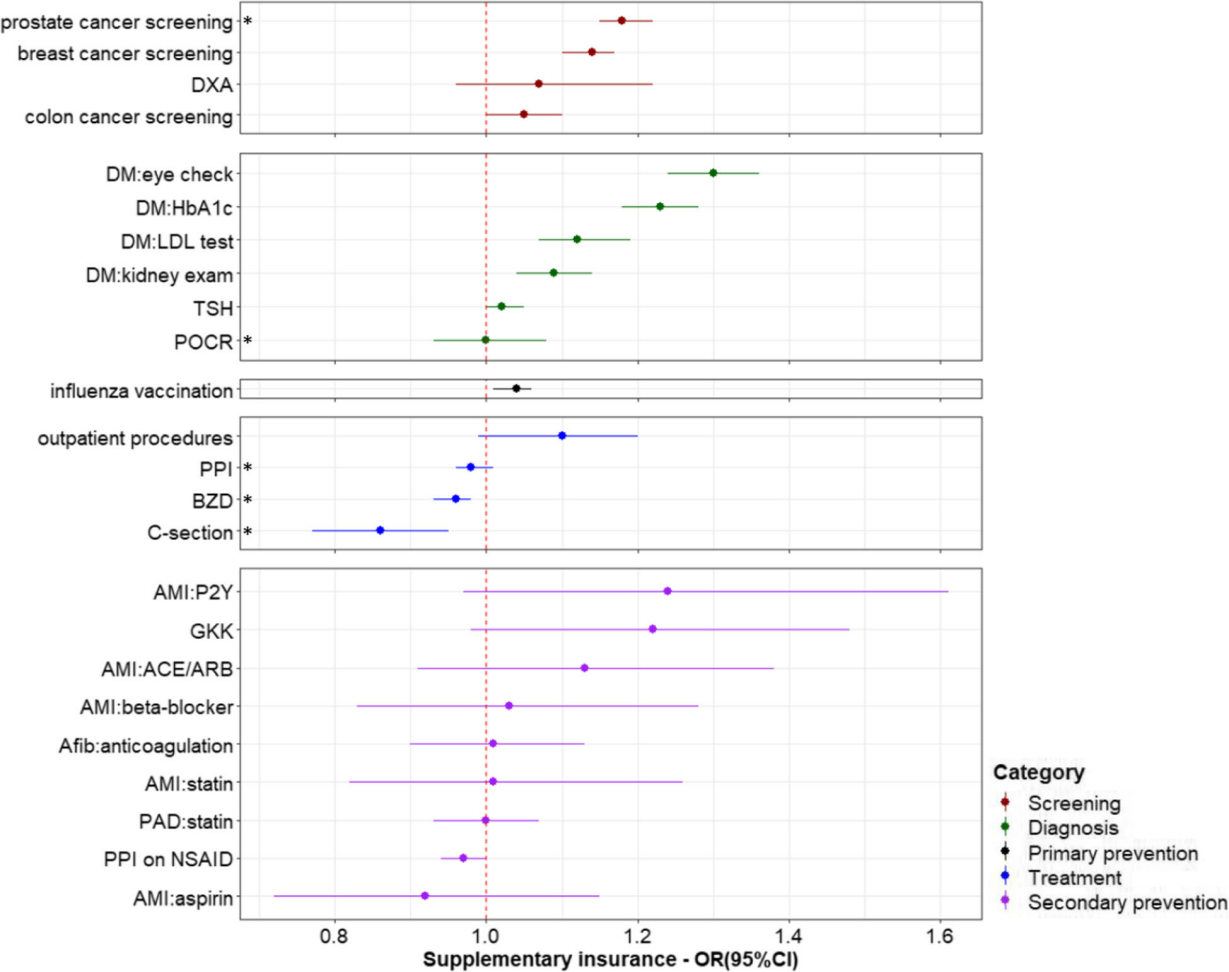
Effects of supplementary insurance on healthcare services utilization. *Indicates services that are discouraged and therefore an odds ratio < 1 indicates better conformity with recommendations, for all other services, an odds ratio > 1 indicates greater use and better guideline conformity. OR: odds ratio; CI: confidence interval; DM: diabetes mellitus; DXA: Dual-energy x-ray absorptiometry; HbA1c: glycated hemoglobin; LDL: low-density lipoprotein; TSH: thyroid stimulating hormone; POCR: outpatient preoperative chest radiography; BZD: benzodiazepines; PPI: proton pump inhibitor; C-section: Cesarean section; AMI: acute myocardial infarction; ACE: angiotensin converting enzyme; ARB: angiotensin receptor blocker; P2Y: clopidogrel, prasugrel or ticagrelor; NSAID: nonsteroidal anti-inflammatory drug; PAD: peripheral artery disease; Afib: atrial fibrillation; GKK: Glucocorticoid

**Fig. 3:**
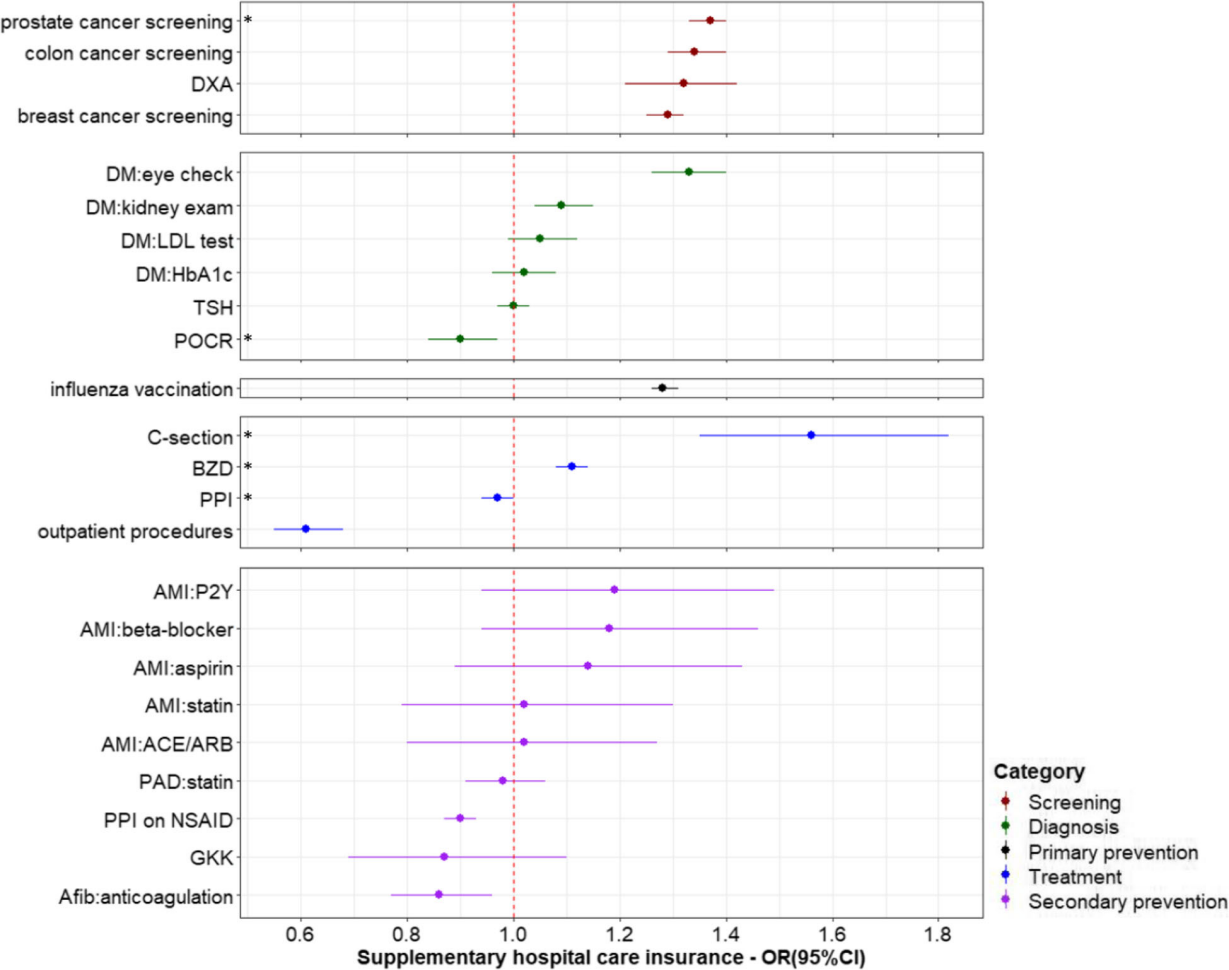
Effects of supplementary hospital insurance on healthcare services utilization. *Indicates services that are discouraged and therefore an odds ratio < 1 indicates better conformity with recommendations, for all other services, an odds ratio > 1 indicates greater use and better guideline conformity. OR: odds ratio; CI: confidence interval; DM: diabetes mellitus; DXA: Dual-energy x-ray absorptiometry; HbA1c: glycated hemoglobin; LDL: low-density lipoprotein; TSH: thyroid stimulating hormone; POCR: outpatient preoperative chest radiography; BZD: benzodiazepines; PPI: proton pump inhibitor; C-section: Cesarean section; AMI: acute myocardial infarction; ACE: angiotensin converting enzyme; ARB: angiotensin receptor blocker; P2Y: clopidogrel, prasugrel or ticagrelor; NSAID: nonsteroidal anti-inflammatory drug; PAD: peripheral artery disease; Afib: atrial fibrillation; GKK: Glucocorticoid

**Fig. 4:**
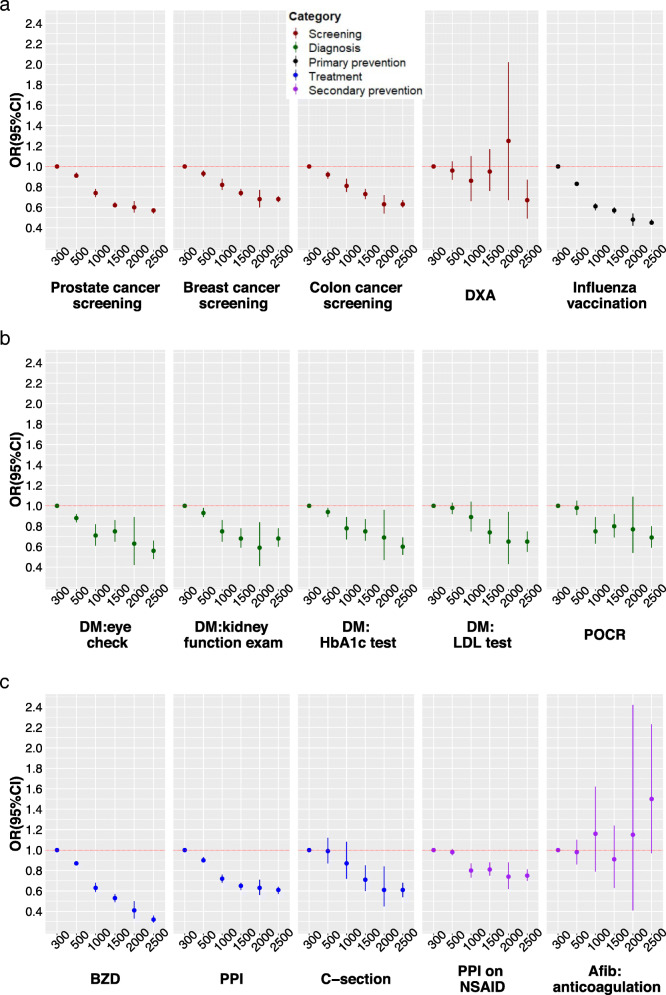
Effects of annual deductible level (Swiss Francs) on healthcare services utilization. OR: odds ratio; CI: confidence interval; DM: diabetes mellitus; DXA: Dual-energy x-ray absorptiometry; HbA1c: glycated hemoglobin; LDL: low-density lipoprotein; TSH: thyroid stimulating hormone; POCR: outpatient preoperative chest radiography; BZD: benzodiazepines; PPI: proton pump inhibitor; C-section: Cesarean section; AMI: acute myocardial infarction; ACE: angiotensin converting enzyme; ARB: angiotensin receptor blocker; P2Y: clopidogrel, prasugrel or ticagrelor; NSAID: nonsteroidal anti-inflammatory drug; PAD: peripheral artery disease; Afib: atrial fibrillation; GKK: Glucocorticoid

The effects of health insurance-related characteristics were mostly consistent across the 24 healthcare services. Having chosen a managed care model was significantly associated with increased use of most services in the categories of screening, diagnosis and secondary prevention, but decreased use of the four services in the treatment category (three of which were not recommended; the fourth were surgical procedures in the outpatient setting) (Fig. 1). The strongest effect was noted for C-section, with an OR of 0.81 (95%CI: 0.73, 0.91). Having any supplementary insurance was associated with increased use of most services (Fig. 2). A negative effect was again seen for C-section, with an OR of 0.86 (95%CI: 0.77, 0.96). Having supplementary hospital insurance was also associated with increased use of most services, including C-section with an OR of 1.58 (95%CI: 1.37, 1.83). People with supplementary hospital insurance were also more likely to undergo surgical procedures with potential for being performed in the outpatient setting, as inpatients (Fig. 3). Related sensitivity analyses showed consistent results. Having a higher deductible was associated with lower utilization of most healthcare services (Fig. 4). All ORs and 95%CIs resulting from the multilevel models for 24 healthcare services are shown in Additional file 1: Supplementary Table 1.

Service-specific factors were associated with healthcare utilization. Patients having surgery in primary, surgical and other specialized hospitals were more likely to receive POCR than patients in central hospitals. Residing in a canton with a coordinated breast cancer screening program was associated with increased mammography utilization with an OR of 1.80 (95%CI: 1.66, 1.97). Associations of ophthalmologist density with eye examinations in diabetes patients with an OR of 1.09 (95%CI: 0.93, 1.23) and of hospital bed density with having surgical procedures in the outpatient setting with an OR of 0.97 (95%CI: 0.94, 1.01)) were non-significant.

### Unadjusted and adjusted regional variation

**Fig. 5:**
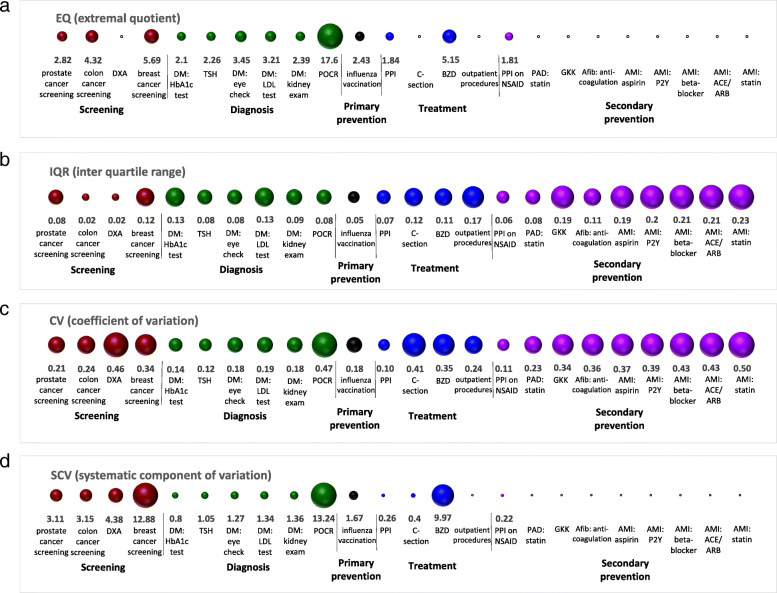
Degree of unadjusted regional variation across 24 selected healthcare services. The blank circle means that for some services, EQ and SCV are not applicable due to insufficient study population. DM: diabetes mellitus; DXA: Dual-energy x-ray absorptiometry; HbA1c: glycated hemoglobin; LDL: low-density lipoprotein; TSH: thyroid stimulating hormone; POCR: outpatient preoperative chest radiography; BZD: benzodiazepines; PPI: proton pump inhibitor; C-section: Cesarean section; AMI: acute myocardial infarction; ACE: angiotensin converting enzyme; ARB: angiotensin receptor blocker; P2Y: clopidogrel, prasugrel or ticagrelor; NSAID: nonsteroidal anti-inflammatory drug; PAD: peripheral artery disease; Afib: atrial fibrillation; GKK: Glucocorticoid

Figure 5 illustrates the degree of unadjusted regional variation across 24 healthcare services; full numerical results are presented in Additional file 2: Supplementary Table 2. The SCVs of six selected healthcare services were above three, indicating that relevant regional variation in the utilization of these services. Among them, the SCVs for POCR (13.24), breast cancer screening (12.88), and long-term benzodiazepine use in older people (9.97) were around or above ten, indicating large regional variation.

**Fig. 6:**
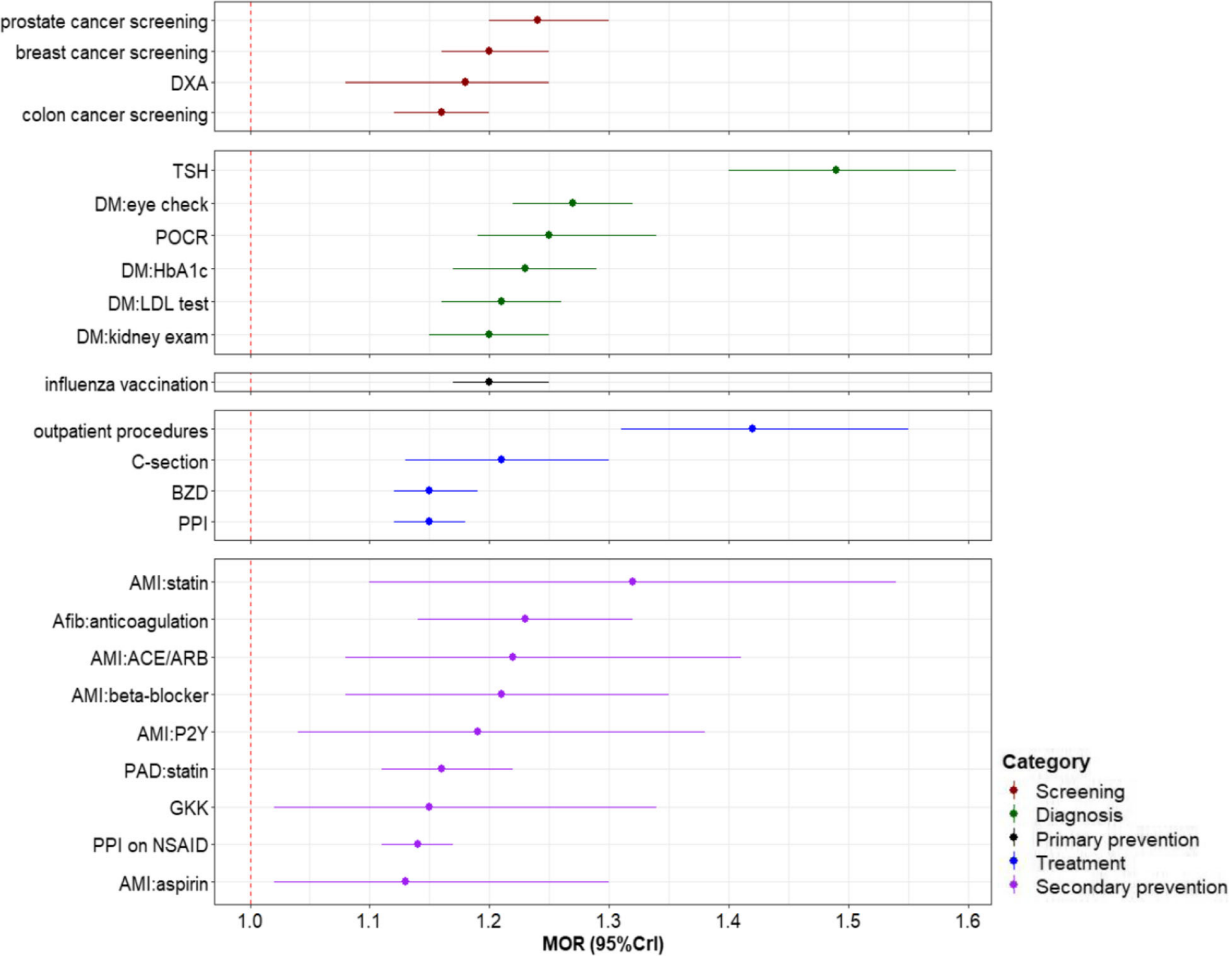
Degree of adjusted regional variation across 24 selected healthcare services. MOR: median odds ratio; CrI: credible interval; DXA: Dual-energy x-ray absorptiometry; TSH: thyroid stimulating hormone; DM: diabetes mellitus; POCR: outpatient preoperative chest radiography; HbA1c: glycated hemoglobin; LDL: low-density lipoprotein; C-section: Cesarean section; BZD: benzodiazepines; PPI: proton pump inhibitor; AMI: acute myocardial infarction; Afib: atrial fibrillation; ACE: angiotensin converting enzyme; ARB: angiotensin receptor blocker; P2Y: clopidogrel, prasugrel or ticagrelor; PAD: peripheral artery disease; GKK: Glucocorticoid; NSAID: nonsteroidal anti-inflammatory drug

Figure 6 shows the degree of unexplained regional variation after controlling for the available influencing factors through multilevel modelling. MORs were below 1.5 for all selected services, and mostly below 1.3. The biggest MOR, close to 1.5, was found for TSH testing, and the smallest MOR, which was just above 1.1, was found in taking aspirin for secondary prevention of AMI. VPCs for all 24 services were within 5.0%, and mostly below 2.0% (Additional file 2:Supplementary Table 2). The combination of relatively small MORs and VPCs implied small unexplained variation for all 24 services. The MOR for POCR in the cross-classified model was 1.25 versus 1.46 in sensitivity analysis, suggesting that some variation among MS regions was accounted for by considering the hospital level.

We detected significant spatial dependence (indicated by significant global Moran’s I value) in raw utilization rates across MS regions for 16 selected healthcare services. After multivariable adjustment through multilevel modelling, global Moran’s I values in model residuals generally decreased, and significant, moderate spatial dependence was detected for only six healthcare services. (Additional file 2: Supplementary Table 2).

## Discussion

We studied 24 diverse healthcare services recommended or discouraged for target populations in clinical guidelines, mainly for major chronic diseases. Overall utilization rates varied substantially, and suggested suboptimal utilization for many services. After controlling for multiple influencing factors, the unexplained regional variation was generally small. Associations between health insurance-related characteristics and utilization were mostly consistent; associations with other influences were rather service-specific.

Although there are no “appropriate” or “optimal” utilization rates known for many healthcare services, strongly recommended services supported by sound evidence may be considered as effective care, and expected to be highly utilized in eligible populations. For example, the studied tests for diabetes complications and secondary prevention medications for AMI patients would fall into this category. Utilization rates for these services between 34 and 70% indicated suboptimal utilization. The utilization rates of healthcare services discouraged in clinical guidelines were generally low as expected. It was also noted that cancer screening rates were apparently very low, presumably because their performance is not recommended annually and we only analysed data from 2014. More accurate estimates can be achieved if considering the recommended screening intervals [31].

The effects of explanatory variables reflected, to some extent, barriers to and facilitators of access to care. In particular, we found coherent associations with health insurance-related characteristics, in a setting with mandatory insurance and quasi-universal access. The data indicated a negative dose-response effect of deductible level on utilization. People with higher deductibles tend to be healthier and willing to take more risks, and some of their invoices may be missed, which may partially explain this observation. However, higher out-of-pocket costs may also make people more reluctant to use services, constituting a financial barrier [32]. While non-insurance practically does not occur in Switzerland, foregoing healthcare utilization due to out-of-pocket costs has been previously documented [33–35]. People having supplementary insurance in addition to mandatory insurance may be wealthier, and more health-conscious and educated on average. Thus, they may seek, or be willing to accept more care, as we observed for most services. Having supplementary hospital insurance was in general associated with increased utilization of healthcare services. This effect was especially prominent in the case of C-section, which is, to a large extent, a preference-sensitive service [36]. Expectedly, having supplementary hospital insurance also made it more likely for patients to receive specific surgical procedures, recommended to be performed on an outpatient basis, as inpatients Enrolees in managed care models were more likely to use healthcare in two thirds of the studied services, which were mostly recommended ones. This may be partially explained by more health awareness. It may also imply that managed care models provide better coordinated and more guideline-concordant care.

Associations between socio-demographics and healthcare utilization were largely service specific. Effects of language region were not consistent, which may be due to different culture and norms, regional health intervention programs, and different practice styles of healthcare providers [37, 38].

People with more comorbidities were generally more likely to use healthcare services. Worse health may trigger more awareness of health-related issues and more contact with healthcare providers, leading to further care. Exceptions were secondary prevention medications in AMI patients and oral anticoagulation in atrial fibrillation patients. Previous studies also reported that more comorbidities were associated with poor adherence to related recommendations [39, 40].

SAVA detected six healthcare services with SCV values over three, among which breast cancer screening, POCR, and long-term use of benzodiazepines in older people had SCVs around ten, suggesting large regional variation. However, after adjusting for available influencing factors, all MORs were relatively small (1.14–1.49). Together with VPCs below 5%, this indicated that the unexplained regional variation in utilization of all considered services was small [24]. The largest unexplained variation was found for TSH testing and surgical procedures performed in the outpatient setting. Both represent preference-sensitive care and decision-making may strongly depend on physicians’ preferences and clinical opinions. Only few previous studies have comprehensively assessed and compared variation in utilization across multiple healthcare services, with mixed results. One study reported moderate variation with MORs between 1.27 to 1.74 for some diabetes-related primary care services [7]. Another study reported large variation with MORs between 2.3 to 21.5 for intensive care unit (ICU) use after 13 major surgical procedures across hospitals [6].

In addition to relatively small regional variation across 24 healthcare services, we found moderate spatial autocorrelation, that is, spatial dependence in the unexplained regional variation in utilization for several healthcare services. Further research could assess the spatial clustering of such regional variation, to explore potential overarching patterns across services, and possibly identify regions with generally superior or inferior performance in terms of appropriate healthcare utilization. This might provide valuable insights for local healthcare intervention and promotion programs.

### Strengths and limitations

Our study has a number of strengths. First, we used a large dataset representing all regions of Switzerland, resulting in large sample sizes for most of the studied healthcare services. Second, we assessed multiple, diverse services, enabling comparison and a broader perspective. The health insurance claims data used provided detailed information on individual insurance-related characteristics, allowing in-depth analyses. Finally, we performed multilevel multivariable modelling for efficient control of confounding.

Several limitations should be considered. First, our selection of healthcare services and eligible populations was not entirely based on burden of disease criteria, mainly because of limitations dictated by the characteristics of Swiss claims data. Second, clinical information is limited in the claims data; outpatient diagnoses are lacking. This may have led to a certain extent of misclassification of eligibility for and utilization of services. Third, we used claims data from a single insurer. Enrolees of other health insurers may theoretically have different characteristics and patterns of healthcare utilization. However, the claims data were based on 1.2 million people from all regions in Switzerland. The benefit package of the mandatory insurance is federally defined and identical for all health insurers. Thus, we expect little deviation of enrollees’ characteristics compared to the whole Swiss population, and the results should essentially be generalizable to the entire country. Fourth, we cannot exclude high variation across different types of units, e.g. healthcare providers for whom we had no detailed information. Finally, the possibility of endogeneity in the relationship between health insurance-related factors and healthcare utilization could not be ruled out in our study. The health status and utilization of healthcare services could to a certain degree impact the choice of health insurance status, and therefore the observed insurance effects might be biased. However, previous studies tried to deal with endogeneity by implementing instrumental variable approaches. They found that the effects of insurance status increased in comparison with analyses non-instrumented on both healthcare utilization and health status [41, 42]. While we are unable to provide a formal assessment, analyses not addressing endogeneity may even underestimate health insurance effects.

## Conclusions

Our study is the first to collectively evaluate regional variation in the utilization of diverse healthcare services and related influencing factors, with a particular focus on insurance-related characteristics. Regional variation in utilization that remained unexplained after multivariable adjustment was relatively small, implying only limited local variation. The consistent effects of health insurance-related factors on healthcare utilization and variation are worth special attention. Despite remaining uncertainties, they suggest that healthcare utilization might be further optimized and a better-performed healthcare system might potentially be achieved through adjustment of health insurance design. For instance, managed care models, which seemed to offer better coordinated, more guideline-adherent care, could be further financially encouraged. Rules regarding annual deductible levels could also be considered to be adjusted, especially for certain types of effective care with strong medical evidence, in order to reduce financial barriers to access. Our comprehensive approach aids in the identification of regional variation and influencing factors of healthcare services use in Switzerland as well as comparable settings worldwide.

## Supplementary Information

**Additional file 1: Table S1.** Effects (OR and 95% CI) of explanatory variables on 24 selected healthcare services utilization in multilevel models.

**Additional file 2: Table S2.** Regional variation in utilization across 24 selected healthcare services.

**Additional file 3: Figure S1.** Relationship between age and healthcare services utilization.

## Data Availability

The data underlying this study cannot be shared publicly because they are the property of Helsana (https://www.helsana.ch/en/helsana-group), and have restricted public access on grounds of patient privacy. The data are managed by Helsana and subsets of the database are available for researchers after request and under specific conditions. Data are available from Helsana (gesundheitskompetenz@helsana.ch) for researchers who meet the criteria for access to confidential data. Helsana will consider the possibilities of the research proposal and decide to grant access if the research questions can be answered with use of the Helsana data. When requests are granted, data are accessible only in a secure environment.

## Abbreviations

SAVA: Small area variation analysis
POCR: Preoperative chest radiography
MS: Mobilité spatiale
EQ: Extremal quotient
IQR: Interquartile range
CV: Coefficient of variation
SCV: Systematic component of variation
OR: Odds ratio
CI: Confidence interval
MOR: Median odds ratio
VPC: Variance partition coefficient
DMARD: Disease-modifying anti-rheumatic drug
TSH: Thyroid-stimulating hormone
AMI: Acute myocardial infarction
PPI: Proton pump inhibitor
ICU: Intensive care unit
