# Supplementary material for: Degree of regional variation and effects of health insurance-related factors on the utilization of 24 diverse healthcare services - a cross-sectional study
Source: BMC Health Serv Res. 2020 Nov 27;20:1091. doi: 10.1186/s12913-020-05930-y (PMC7694910; doi:10.1186/s12913-020-05930-y)
Supplement: Supplementary file 1 — Additional file 1: Table S1. Effects (OR and 95% CI) of explanatory variables on 24 selected healthcare services utilization in multilevel models. [file 12913_2020_5930_MOESM1_ESM.docx]

Supplementary Table 1. Effects (OR and 95% CI) of explanatory variables on 24 selected healthcare services utilization in multilevel models.

| Healthcare service | Age | Female gender | Urban | Purchasing power index | Language region* | | Number of comorbidities | | |
| --- | --- | --- | --- | --- | --- | --- | --- | --- | --- |
|  |  |  |  |  | French | Italian | 1 | 2 | >2 |
| Colon cancer screening | - | 0.93(0.90,0.96) | 1.07(1.02,1.12) | 1.25(1.16,1.38) | 0.86(0.79,0.94) | 1.11(0.98,1.27) | 1.30(1.24,1.36) | 1.31(1.25,1.38) | 1.45(1.38,1.52) |
| Breast cancer screening | - | - | 1.06(1.03,1.11) | 1.09(1.01,1.18) | 1.65(1.50,1.81) | 1.69(1.43-2.01) | 1.26(1.22-1.30) | 1.30(1.25-1.35) | 1.30(1.25,1.34) |
| Prostate cancer screening | - | - | 1.10(1.07,1.14) | 1.20(1.12-1.28) | 1.38(1.25,1.53) | 1.50(1.28-1.74) | 1.66(1.61-1.71) | 1.90(1.84-1.95) | 1.83(1.79,1.89) |
| Osteoporosis screening | - | 3.66(3.10,3.99) | 1.15(1.04,1.27) | 1.23(1.01,1.44) | - | - | - | - | - |
| DM: HbA1c test | - | 0.96(0.93,1.00) | 0.96(0.90,1.02) | 0.88(0.78,0.98) | 0.51(0.46,0.58) | 0.48(0.39,0.56) | 1.10(1.01,1.20) | 1.20(1.10,1.30) | 1.24(1.14,1.35) |
| DM: eye check | - | 1.16(1.12,1.20) | - | 1.16(0.99,1.33) | 0.83(0.74,0.95) | 0.78(0.64,0.94) | 0.95(0.88,1.03) | 1.08(1.00,1.17) | 1.24(1.15,1.33) |
| DM: kidney exam | - | 1.07(1.04,1.22) | 1.14(1.09,1.20) | 1.06(0.94,1.21) | 1.18(1.07,1.31) | 1.53(1.27,1.85) | 1.06(0.98,1.14) | 1.21(1.13,1.29) | 1.38(1.29,1.47) |
| DM: LDL test | - | 0.89(0.85,0.93) | 1.12(1.05,1.19) | - | 2.12(1.90,2.40) | 1.40(1.16,1.71) | 1.17(1.08,1.27) | 1.45(1.33,1.56) | 1.57(1.45,1.70) |
| TSH | - | 0.79(0.77,0.81) | 0.97(0.93,1.02) | - | 0.91(0.78,1.08) | 1.12(0.80,1.39) | - | - | - |
| POCR | 1.03(1.02,1.05) | 0.84(0.80,0.89) | - | - | - | - | 1.04(0.93,1.15) | 1.10(0.99,1.23) | 1.16(1.04,1.30) |
| Influenza vaccination | - | 0.86(0.84,0.87) | 1.04(1.02,1.07) | 1.14(0.99,1.34) | 1.03(0.92,1.15) | 1.03(0.87,1.20) | 1.75(1.70,1.81) | 2.53(2.46,2.60) | 4.21(4.09,4.32) |
| BZD | - | 1.96(1.89,2.00) | - | - | 2.00(1.85,2.13) | 2.17(1.92,2.38) | 1.92(1.85,2.04) | 2.63(2.50,2.78) | 4.00(3.85,4.17) |
| PPI | - | 0.80(0.78,0.82) | 1.00(0.96,1.03) | 0.88(0.83,0.94) | 1.10(1.02,1.19) | 0.95(0.85,1.09) | 1.35(1.31,1.41) | 1.72(1.67,1.79) | 3.23(3.13,3.33) |
| Outpatient procedures | - | 1.98(1.82,2.16) | 0.97(0.85,1.10) | 0.71(0.53,0.89) | 2.69(2.08,3.59) | 0.93(0.62,1.29) | - | - | - |
| C-section | 1.05(1.04,1.06) | - | - | - | 0.73(0.61,0.87) | 0.79(0.59,1.02) | 1.27(1.15,1.41) | 1.40(1.20,1.63) | 2.14(1.66,2.71) |
| AMI: aspirin | 0.97(0.96,0.98) | 1.08(0.88,1.31) | - | - | - | - | 0.61(0.41,0.88) | 0.33(0.23,0.45) | 0.25(0.18,0.34) |
| AMI: statin | - | 0.88(0.70,1.10) | - | - | 0.61(0.43,0.85) | 0.74(0.48,1.10) | 0.57(0.38,0.82) | 0.41(0.29,0.56) | 0.31(0.23,0.42) |
| AMI: beta-blocker | 0.98(0.97,0.99) | 0.99(0.79,1.20) | - | 0.50(0.30,0.79) | - | - | 0.86(0.58,1.24) | 0.63(0.44,0.87) | 0.59(0.43,0.79) |
| AMI: ACE/ARB | 0.98(0.97,0.98) | 1.00(0.82,1.23) | - | - | - | - | 0.73(0.50,1.00) | 0.62(0.44,0.83) | 0.55(0.41,0.73) |
| AMI: P2Y | - | 0.85(0.69,1.04) | - | - | 0.97(0.71,1.25) | 1.48(1.02,2.02) | 0.65(0.43,0.95) | 0.55(0.38,0.78) | 0.53(0.37,0.72) |
| PPI with NSAID | - | 1.05(1.02,1.07) | - | - | 0.90(0.83,0.97) | 1.03(0.91,1.18) | 0.97(0.92,1.01) | 1.07(1.02,1.12) | 1.60(1.54,1.66) |
| PAD: statin | - | 0.52(0.49,0.55) | 0.95(0.87,1.05) | - | 0.74(0.66,0.83) | 1.14(0.93,1.38) | 1.42(1.12,1.79) | 4.20(3.45,5.14) | 6.72(5.58,8.11) |
| Afib: anticoagulation | - | 1.16(1.04,1.29) | - | - | 0.99(0.81,1.19) | 1.28(0.97,1.64) | 1.01(0.70,1.45) | 0.85(0.60,1.20) | 0.70(0.51,0.98) |
| GKK | 1.01(1.01,1.02) | 0.82(0.67,1.02) | - | - | 0.54(0.40,0.72) | 1.41(0.92,2.05) | 1.28(0.94,1.70) | 1.46(1.06,1.96) | 1.40(1.07,1.81) |

Continued Supplementary Table 1

| Healthcare service | Supplementary insurance | Managed care models | Supplementary hospital insurance | Annual deductible level (Swiss Francs) | | | | |
| --- | --- | --- | --- | --- | --- | --- | --- | --- |
|  |  |  |  | 500 | 1000 | 1500 | 2000 | 2500 |
| Colon cancer screening | 1.05(1.00,1.10) | 1.12(1.08,1.15) | 1.34(1.29,1.40) | 0.92(0.88,0.95) | 0.81(0.75,0.88) | 0.73(0.68,0.78) | 0.63(0.54,0.72) | 0.63(0.60,0.67) |
| Breast cancer screening | 1.14(1.10-1.17) | 1.13(1.10-1.16) | 1.29(1.25-1.32) | 0.93(0.90-0.96) | 0.82(0.77-0.88) | 0.74(0.71-0.78) | 0.68(0.60-0.77) | 0.68(0.65-0.71) |
| Prostate cancer screening | 1.18(1.14-1.22) | 1.13(1.11-1.15) | 1.36(1.33-1.40) | 0.91(0.89-0.94) | 0.74(0.70-0.78) | 0.62(0.60-0.65) | 0.60(0.55-0.66) | 0.56(0.54-0.59) |
| Osteoporosis screening | 1.07(0.96,1.22) | 0.99(0.92,1.06) | 1.32(1.21,1.42) | 0.96(0.87,1.05) | 0.86(0.66,1.10) | 0.95(0.76,1.17) | 1.25(0.67,2.02) | 0.67(0.49,0.87) |
| DM: HbA1c test | 1.23(1.18,1.28) | 1.13(1.08,1.17) | 1.02(0.96,1.08) | 0.94(0.89,0.98) | 0.78(0.67,0.89) | 0.75(0.66,0.87) | 0.69(0.47,0.96) | 0.60(0.52,0.69) |
| DM: eye check | 1.30(1.24,1.36) | 1.19(1.14,1.23) | 1.33(1.26,1.40) | 0.88(0.84,0.92) | 0.71(0.61,0.82) | 0.75(0.65,0.86) | 0.63(0.42,0.89) | 0.56(0.48,0.66) |
| DM: kidney exam | 1.09(1.04,1.14) | 1.05(1.01,1.09) | 1.09(1.04,1.15) | 0.93(0.89,0.98) | 0.75(0.65,0.86) | 0.68(0.59,0.78) | 0.59(0.41,0.84) | 0.68(0.60,0.78) |
| DM: LDL test | 1.12(1.07,1.19) | 1.08(1.02,1.13) | 1.05(0.99,1.12) | 0.98(0.92,1.03) | 0.89(0.75,1.04) | 0.74(0.63,0.87) | 0.65(0.43,0.94) | 0.65(0.55,0.75) |
| TSH | 1.02(1.00,1.05) | 1.07(1.05,1.10) | 1.00(0.97,1.03) | - | - | - | - | - |
| POCR | 1.00(0.93,1.08) | 0.98(0.93,1.04) | 0.90(0.84,0.97) | 0.98(0.91,1.05) | 0.75(0.63,0.89) | 0.80(0.69,0.92) | 0.77(0.54,1.09) | 0.69(0.59,0.80) |
| Influenza vaccination | 1.04(1.01,1.06) | 0.97(0.96,0.99) | 1.28(1.26,1.31) | 0.83(0.81,0.85) | 0.61(0.57,0.64) | 0.57(0.54,0.60) | 0.48(0.42,0.54) | 0.45(0.43,0.48) |
| BZD | 0.96(0.93,0.98) | 0.92(0.90,0.94) | 1.11(1.08,1.14) | 0.87(0.85,0.89) | 0.63(0.59,0.68) | 0.53(0.49,0.57) | 0.41(0.33,0.50) | 0.32(0.29,0.36) |
| PPI | 0.98(0.96,1.01) | 0.88(0.86,0.90) | 0.97(0.94,1.00) | 0.90(0.88,0.93) | 0.72(0.68,0.76) | 0.65(0.61,0.68) | 0.63(0.56,0.71) | 0.61(0.57,0.64) |
| Outpatient procedures | 1.10(0.99,1.20) | 0.95(0.87,1.04) | 0.61(0.55,0.68) | - | - | - | - | - |
| C-section | 0.86(0.77,0.96) | 0.81(0.73,0.91) | 1.58(1.37,1.83) | 0.99(0.87,1.12) | 0.89(0.71,1.08) | 0.72(0.60,0.84) | 0.62(0.45,0.83) | 0.61(0.53,0.70) |
| AMI: aspirin | 0.92(0.72,1.15) | 1.15(0.95,1.38) | 1.14(0.89,1.43) | - | - | - | - | - |
| AMI: statin | 1.01(0.82,1.26) | 1.13(0.92,1.36) | 1.02(0.79,1.30) | - | - | - | - | - |
| AMI: beta-blocker | 1.03(0.83,1.28) | 1.21(1.01,1.44) | 1.18(0.94,1.46) | - | - | - | - | - |
| AMI: ACE/ARB | 1.13(0.91,1.38) | 1.19(0.98,1.41) | 1.02(0.80,1.27) | - | - | - | - | - |
| AMI: P2Y | 1.24(0.97,1.61) | 1.23(1.02,1.49) | 1.19(0.94,1.49) | - | - | - | - | - |
| PPI with NSAID | 0.97(0.94,1.00) | 0.90(0.88,0.93) | 0.90(0.87,0.93) | 0.98(0.95,1.01) | 0.80(0.73,0.87) | 0.81(0.75,0.88) | 0.74(0.62,0.88) | 0.75(0.70,0.81) |
| PAD: statin | 1.00(0.93,1.07) | 0.97(0.90,1.03) | 0.98(0.91,1.06) | - | - | - | - | - |
| Afib: anticoagulation | 1.01(0.90,1.13) | 1.05(0.95,1.17) | 0.86(0.77,0.96) | 0.98(0.86,1.10) | 1.16(0.79,1.62) | 0.91(0.63,1.24) | 1.15(0.41,2.42) | 1.50(0.97,2.23) |
| GKK | 1.22(0.98,1.48) | 0.85(0.69,1.03) | 0.87(0.69,1.10) | - | - | - | - | - |

* With reference to German language region. OR: odds ratio; CI: confidence interval; DM: diabetes mellitus; HbA1c: glycated hemoglobin; LDL: low-density lipoprotein; TSH: thyroid stimulating hormone; POCR: outpatient preoperative chest radiography; BZD: benzodiazepines; PPI: proton pump inhibitor; C-section: Cesarean section; AMI: acute myocardial infarction; ACE: angiotensin converting enzyme; ARB: angiotensin receptor blocker; P2Y: clopidogrel, prasugrel or ticagrelor; NSAID: nonsteroidal anti-inflammatory drug; PAD: peripheral artery disease; Afib: atrial fibrillation; GKK: Glucocorticoid.
