# Supplementary material for: Degree of regional variation and effects of health insurance-related factors on the utilization of 24 diverse healthcare services - a cross-sectional study
Source: BMC Health Serv Res. 2020 Nov 27;20:1091. doi: 10.1186/s12913-020-05930-y (PMC7694910; doi:10.1186/s12913-020-05930-y)
Supplement: Supplementary file 2 — Additional file 2: Table S2. Regional variation in utilization across 24 selected healthcare services. [file 12913_2020_5930_MOESM2_ESM.docx]

Supplementary Table 2. Regional variation in utilization across 24 selected healthcare services.

| Service category | Healthcare service | Unadjusted regional variation | | | | Adjusted regional variation | | Moran’s I (raw rates) | Moran’s I (model residuals) |
| --- | --- | --- | --- | --- | --- | --- | --- | --- | --- |
|  |  | EQ (extremal quotient) | IQR (inter quartile range) | CV (coefficient of variation) | SCV (systematic component of variation) | MOR (95% CrI) in multilevel models | VPC (variance partition coefficient) |  |  |
| Screening | Colon cancer screening | 4.32 | 0.02 | 0.24 | 3.15 | 1.16(1.12,1.20) | 0.71% | 0.222** | 0.084 |
|  | Breast cancer screening | 5.69 | 0.12 | 0.34 | 12.88 | 1.20(1.16,1.25) | 1.13% | 0.622** | 0.070 |
|  | Prostate cancer screening | 2.82 | 0.08 | 0.21 | 3.11 | 1.24(1.20,1.30) | 1.57% | 0.551** | 0.490** |
|  | Osteoporosis screening | na | 0.02 | 0.46 | 4.38 | 1.18(1.08,1.25) | 0.87% | 0.221** | 0.102 |
| Diagnosis | DM: HbA1c test | 2.10 | 0.13 | 0.14 | 0.8 | 1.23(1.17,1.29) | 1.40% | 0.500** | 0.245** |
|  | DM: kidney exam | 2.39 | 0.09 | 0.18 | 1.36 | 1.20(1.15,1.25) | 1.12% | 0.284** | 0.193** |
|  | DM: LDL test | 3.21 | 0.13 | 0.19 | 1.34 | 1.21(1.16,1.26) | 1.20% | 0.547** | 0.239** |
|  | DM: eye check | 3.45 | 0.08 | 0.18 | 1.27 | 1.27(1.22,1.32) | 1.82% | 0.179** | 0.120* |
|  | TSH | 2.26 | 0.08 | 0.12 | 1.05 | 1.49(1.40,1.59) | 5.01% | 0.301** | 0.298** |
|  | POCR | 17.6 | 0.08 | 0.47 | 13.24 | 1.25(1.19,1.34) | 1.64% | 0.263** | 0.066 |
| Primary prevention | influenza vaccination | 2.43 | 0.05 | 0.18 | 1.67 | 1.20(1.17,1.25) | 1.14% | 0.012 | 0.014 |
| Treatment | BZD | 5.15 | 0.11 | 0.35 | 9.97 | 1.15(1.12,1.19) | 0.66% | 0.656** | 0.260** |
|  | PPI | 1.84 | 0.07 | 0.10 | 0.26 | 1.15(1.12,1.18) | 0.63% | 0.199** | 0.073 |
|  | Outpatient procedures | na | 0.17 | 0.24 | negative | 1.42(1.31,1.55) | 3.92% | 0.318** | 0.131* |
|  | C-section | na | 0.12 | 0.41 | 0.41 | 1.21(1.13,1.30) | 1.20% | 0.0227 | 0.070 |
| Secondary prevention | AMI: aspirin | na | 0.19 | 0.37 | negative | 1.13(1.02,1.30) | 0.49% | -0.052 | 0.057 |
|  | AMI: statin | na | 0.23 | 0.50 | negative | 1.32(1.10,1.54) | 2.50% | 0.036 | 0.157* |
|  | AMI: beta-blocker | na | 0.21 | 0.43 | negative | 1.21(1.08,1.35) | 1.16% | 0.134* | 0.097 |
|  | AMI: ACE/ARB | na | 0.21 | 0.43 | negative | 1.22(1.08,1.41) | 1.30% | -0.104 | 0.007 |
|  | AMI: P2Y | na | 0.20 | 0.39 | negative | 1.19(1.04,1.38) | 0.96% | -0.009 | 0.006 |
|  | PPI with NSAID | 1.81 | 0.06 | 0.11 | 0.22 | 1.14(1.11,1.17) | 0.57% | 0.189** | 0.170* |
|  | PAD: statin | na | 0.08 | 0.23 | negative | 1.16(1.11,1.22) | 0.75% | 0.247** | 0.043 |
|  | Afib: anticoagulation | na | 0.11 | 0.36 | negative | 1.23(1.14,1.32) | 1.41% | -0.102 | 0.100 |
|  | GKK | na | 0.19 | 0.34 | negative | 1.15(1.02,1.34) | 0.68% | 0.105 | -0.001 |

*p<0.05; **p<0.01

MOR: median odds ratio; Crl: credible interval; DM: diabetes mellitus; HbA1c: glycated hemoglobin; LDL: low-density lipoprotein; TSH: thyroid stimulating hormone; POCR: outpatient preoperative chest radiography; BZD: benzodiazepines; PPI: proton pump inhibitor; C-section: Cesarean section; AMI: acute myocardial infarction; ACE: angiotensin converting enzyme; ARB: angiotensin receptor blocker; P2Y: clopidogrel, prasugrel or ticagrelor; NSAID: nonsteroidal anti-inflammatory drug; PAD: peripheral artery disease; Afib: atrial fibrillation; GKK: Glucocorticoid; na: not applicable
