# Supplementary material for: Degree of regional variation and effects of health insurance-related factors on the utilization of 24 diverse healthcare services - a cross-sectional study
Source: BMC Health Serv Res. 2020 Nov 27;20:1091. doi: 10.1186/s12913-020-05930-y (PMC7694910; doi:10.1186/s12913-020-05930-y)
Supplement: Supplementary file 3 — Additional file 3: Figure S1. Relationship between age and healthcare services utilization. [file 12913_2020_5930_MOESM3_ESM.docx]

Supplementary Figure 1. Relationship between age and healthcare services utilization.


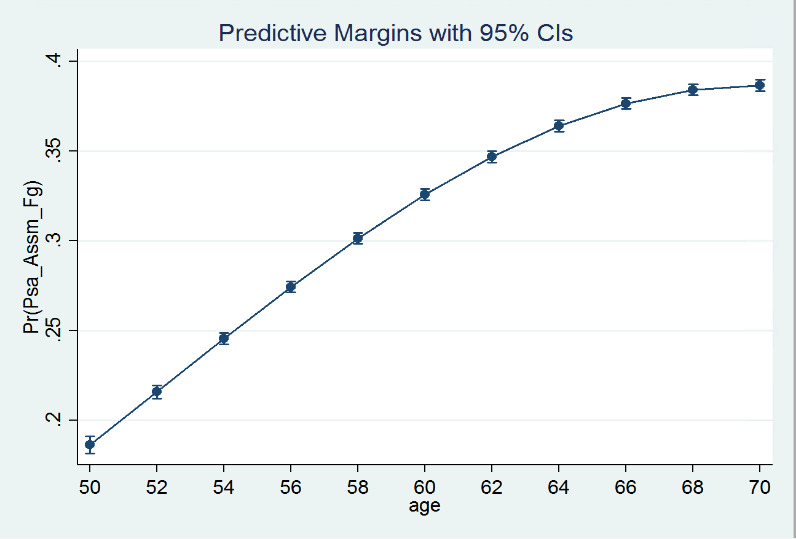
a. Colon cancer screening b. Breast cancer screening c. Prostate cancer screening


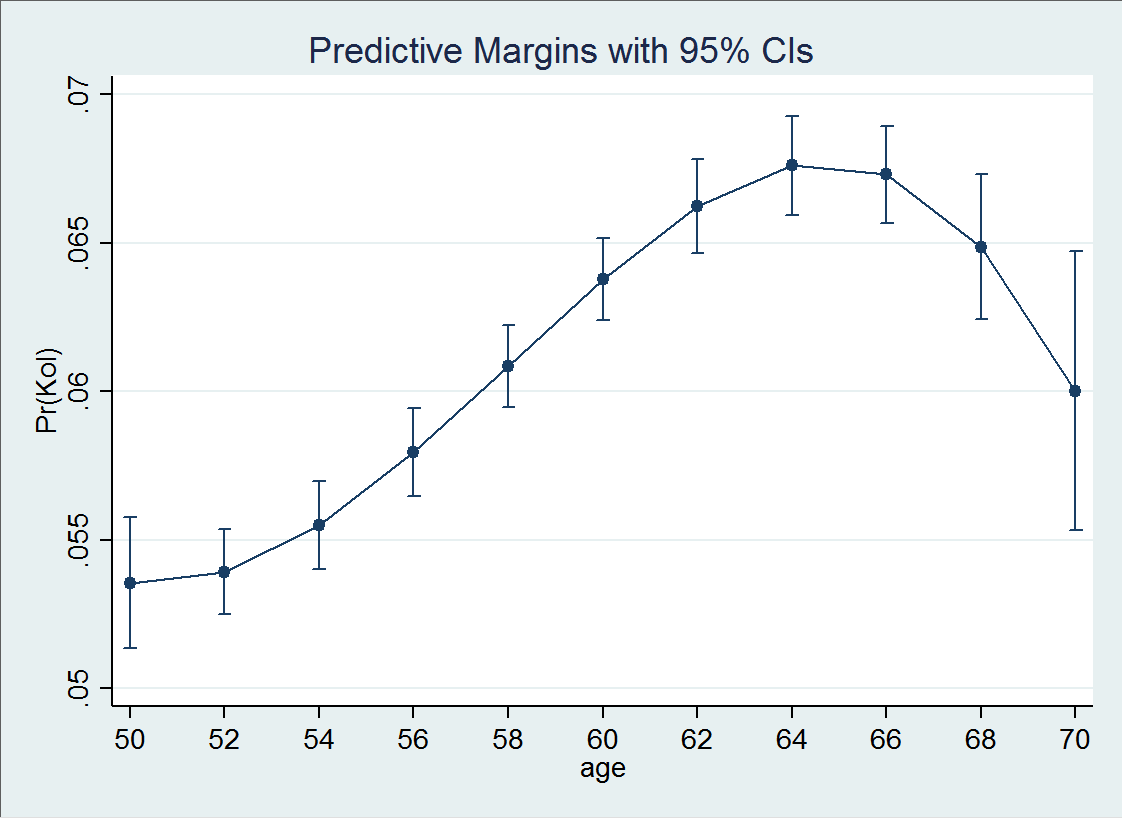

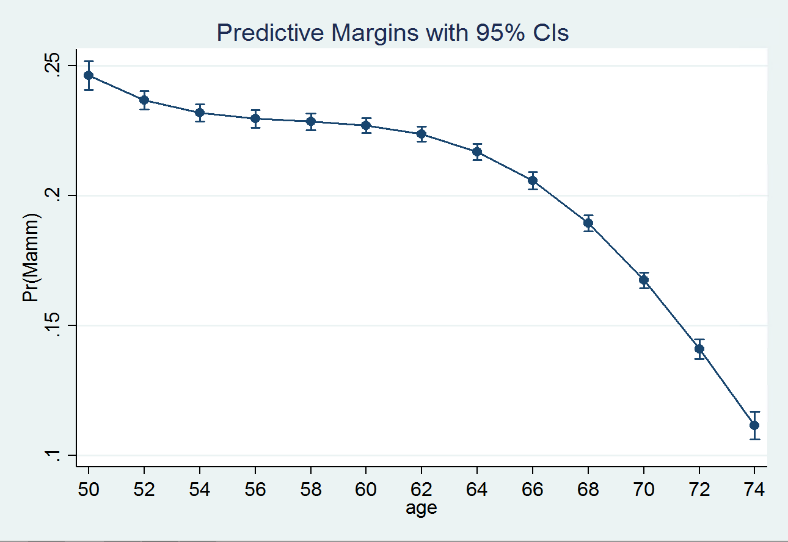


d. Osteoporosis screening e. DM: HbA1c test f. DM: kidney exam


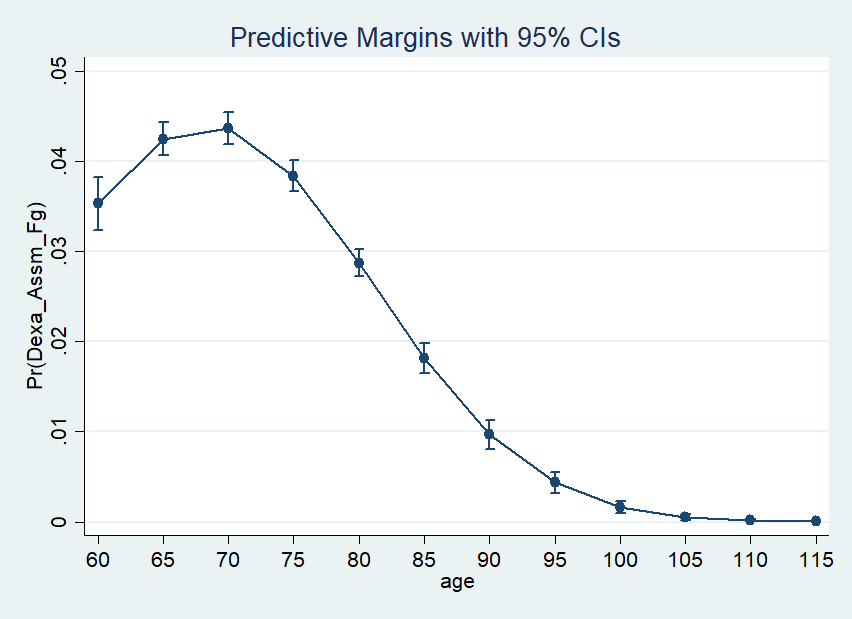

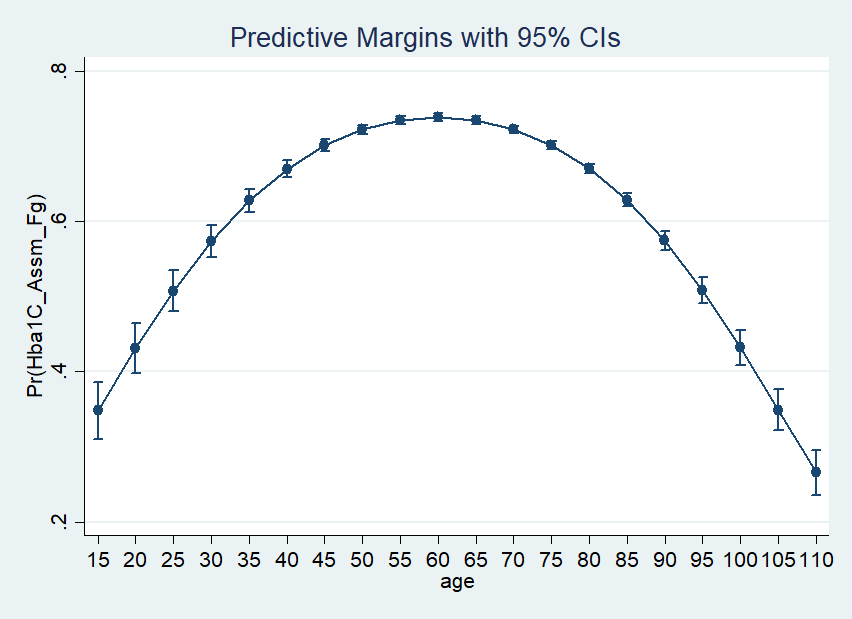

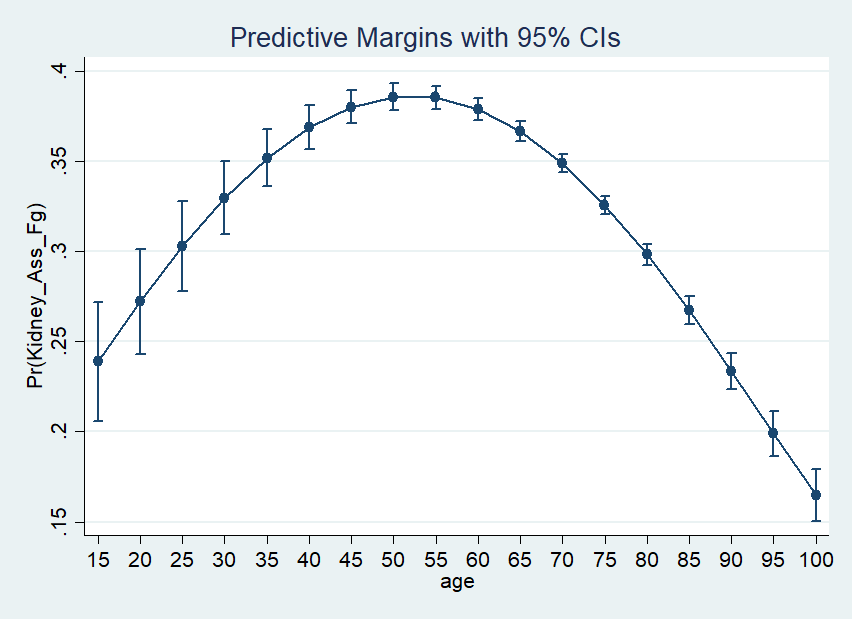


g. DM: LDL test h. DM: eye check i. TSH test


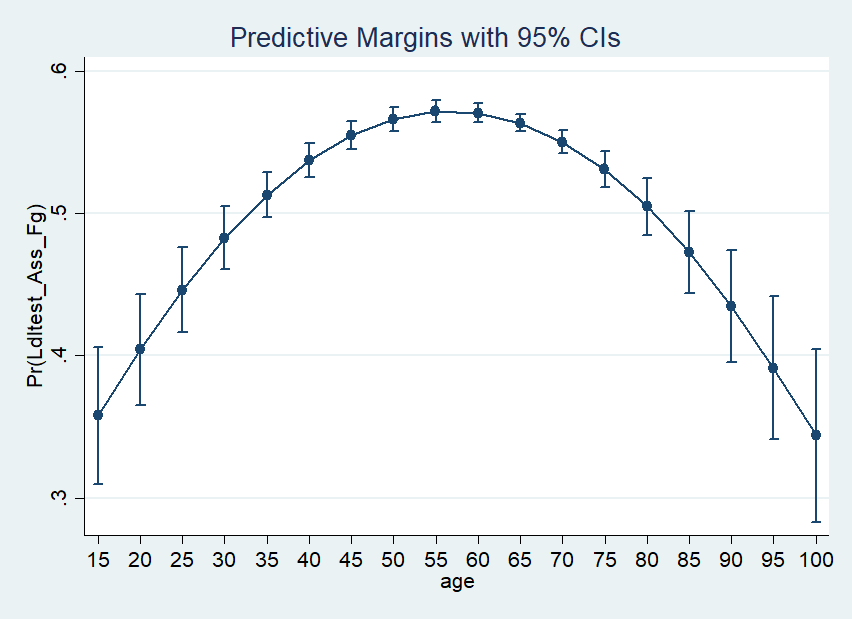

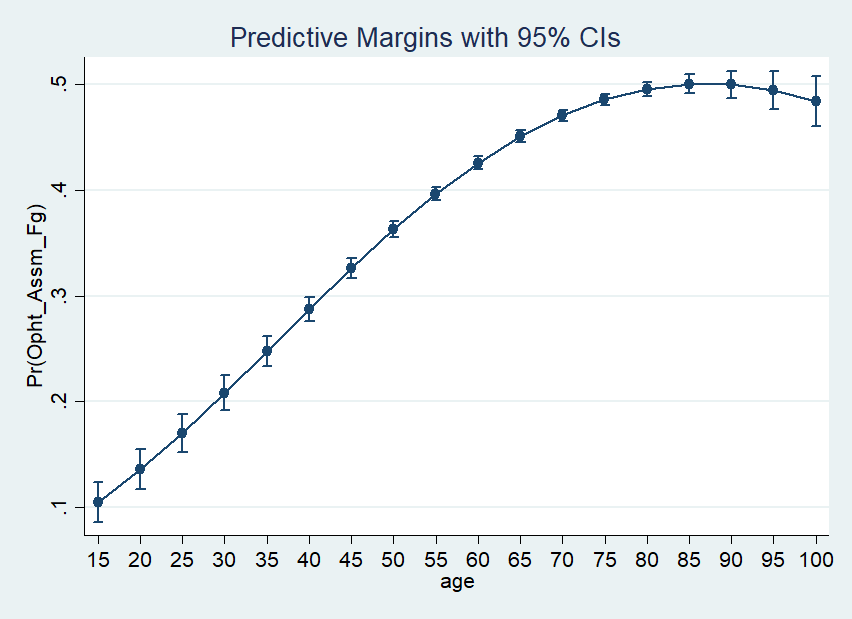

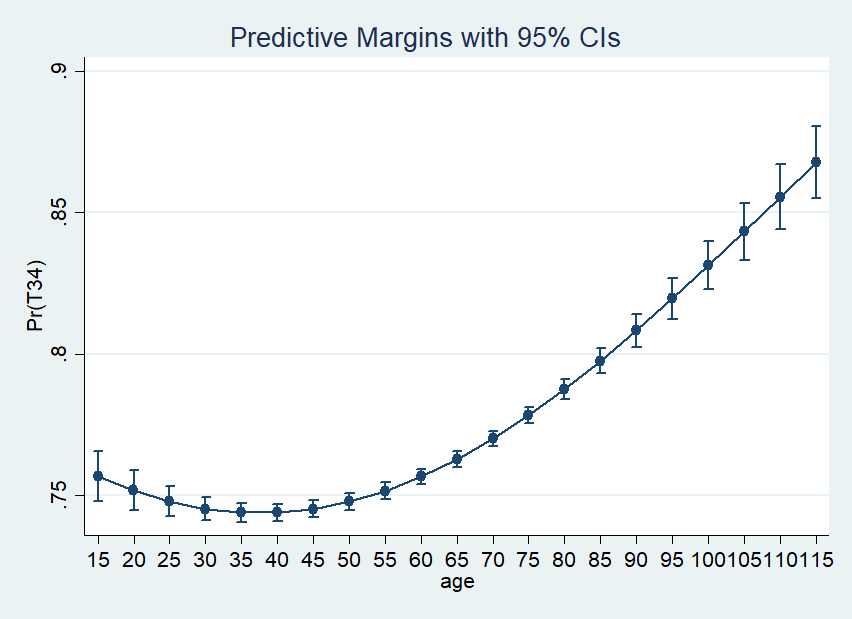


j. Influenza vaccination k. BZD l. PPI


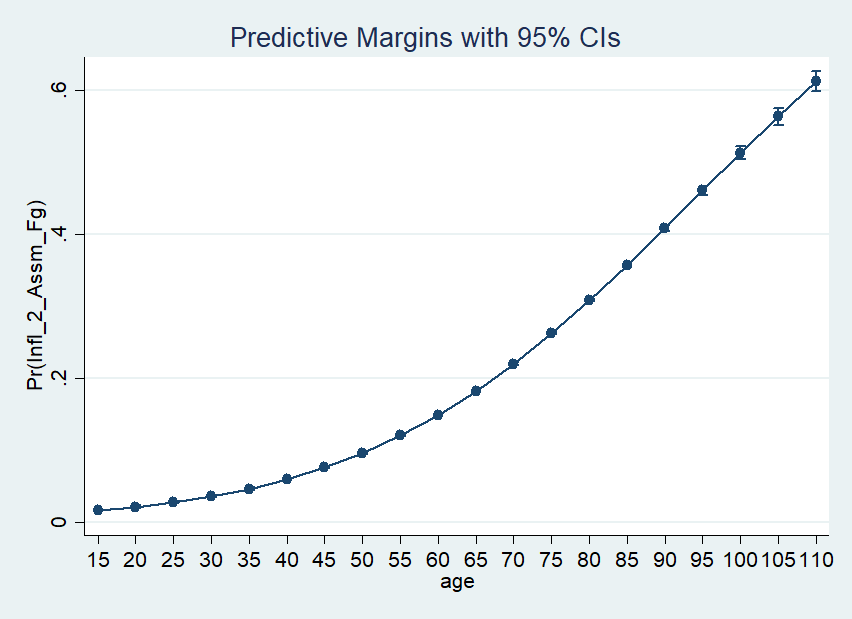

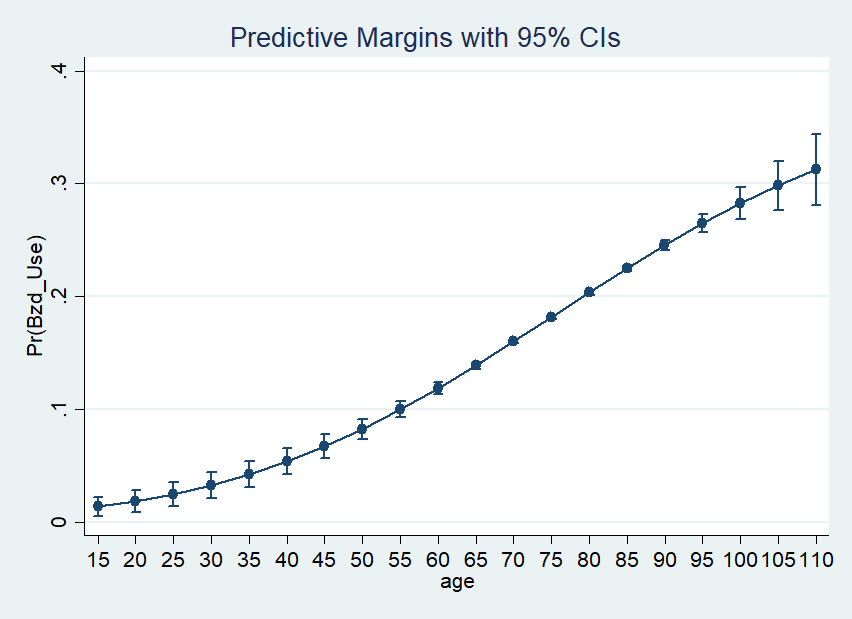

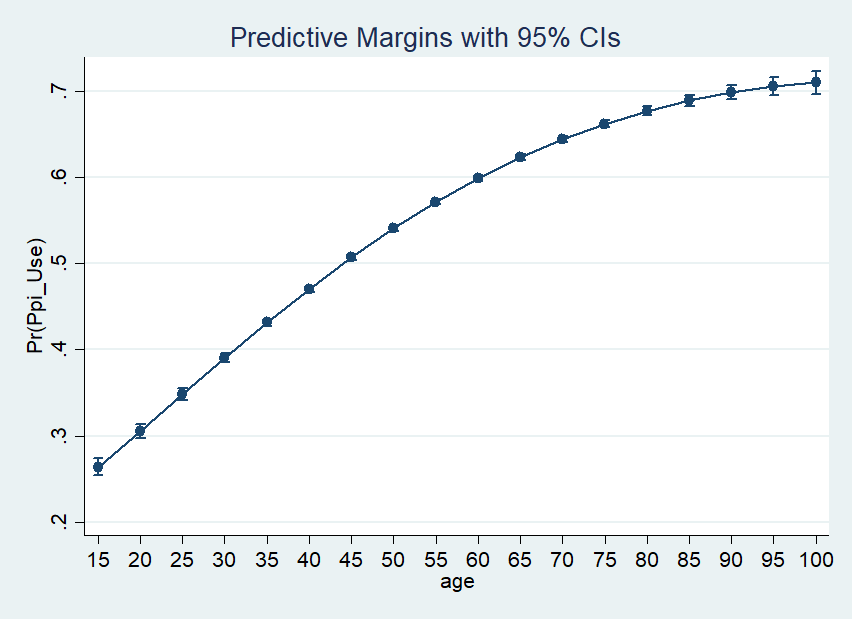


m. Outpatient procedures n. AMI: statin o. AMI: P2Y


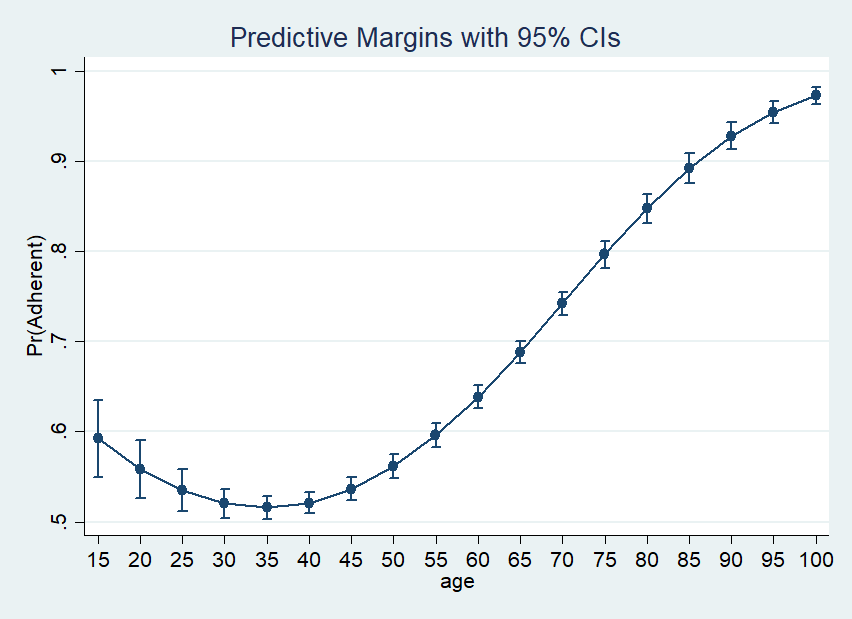

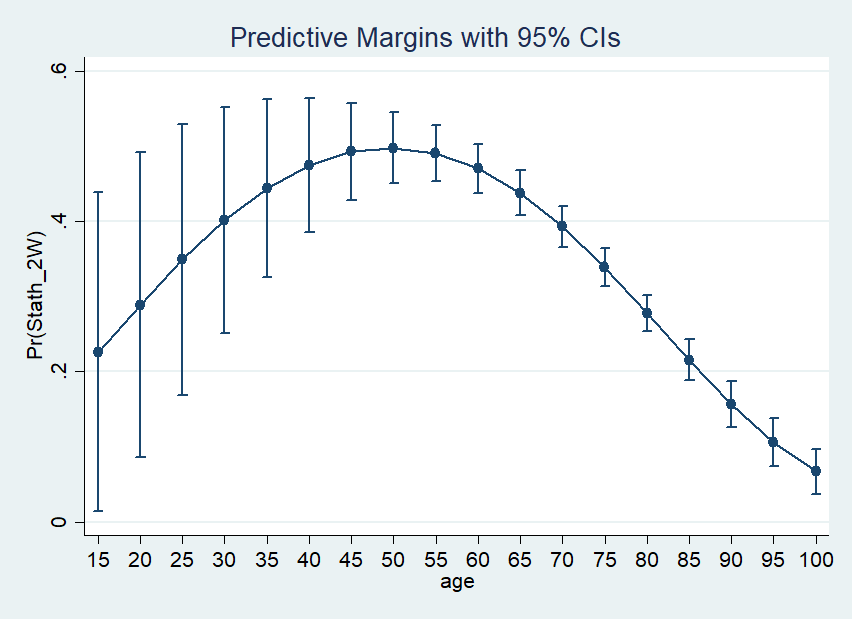

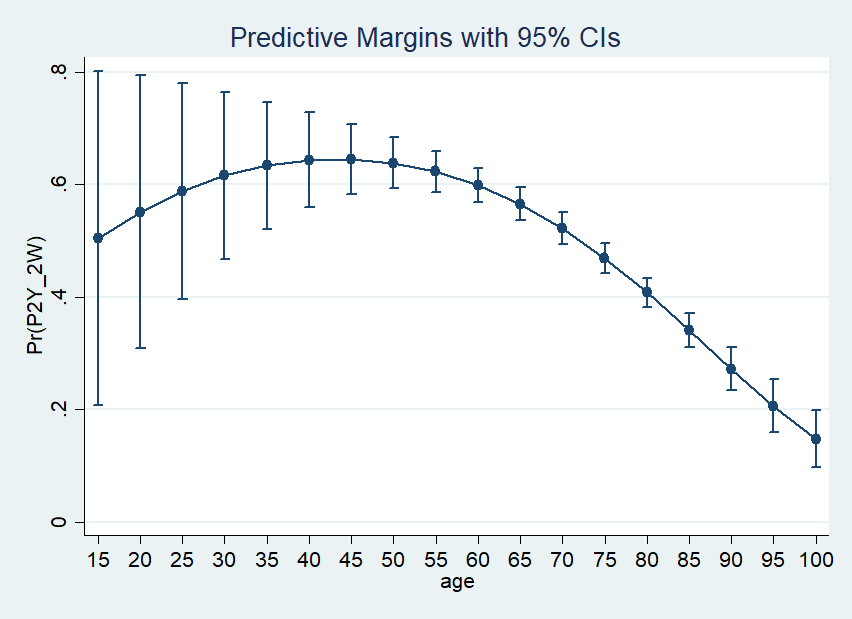


p. PPI with NSAID q. PAD: statin r. Afib: anticoagulation


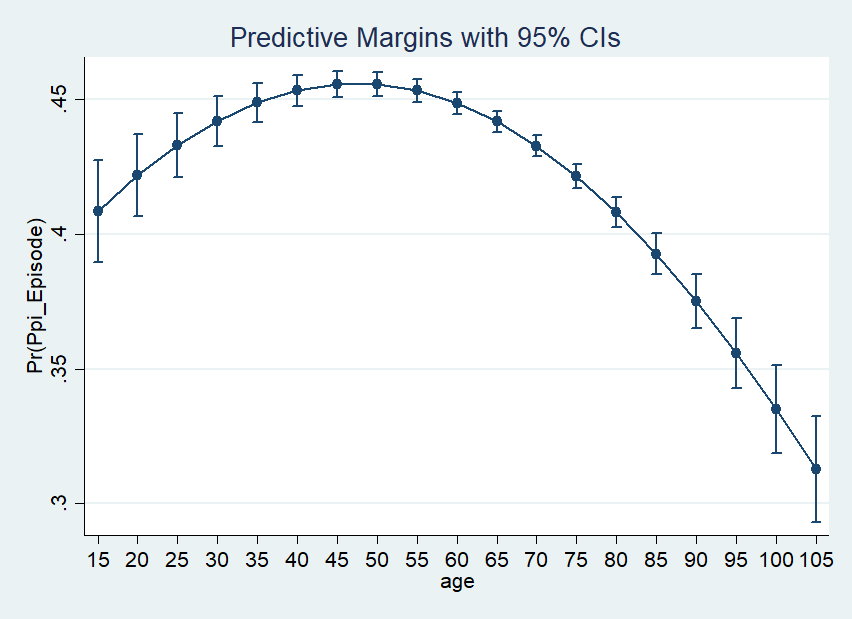

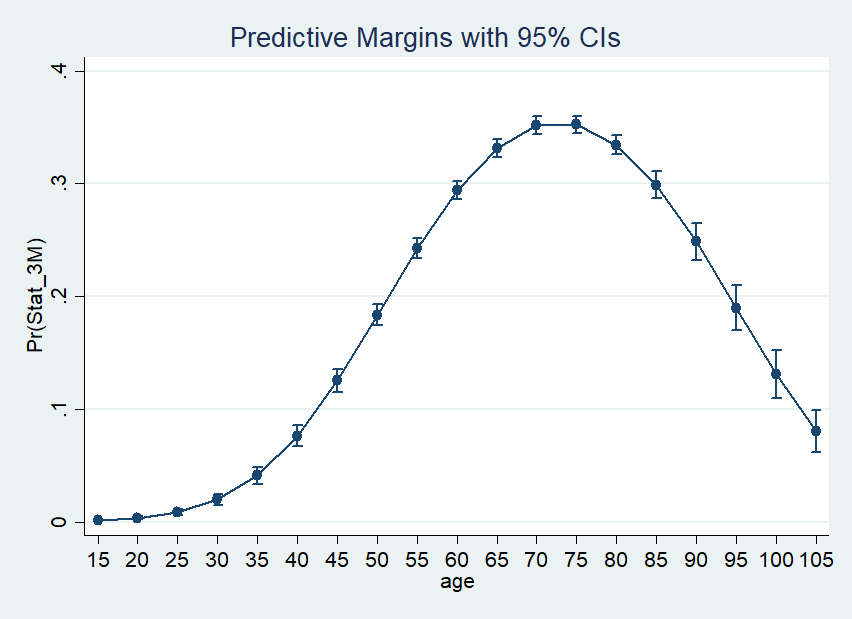

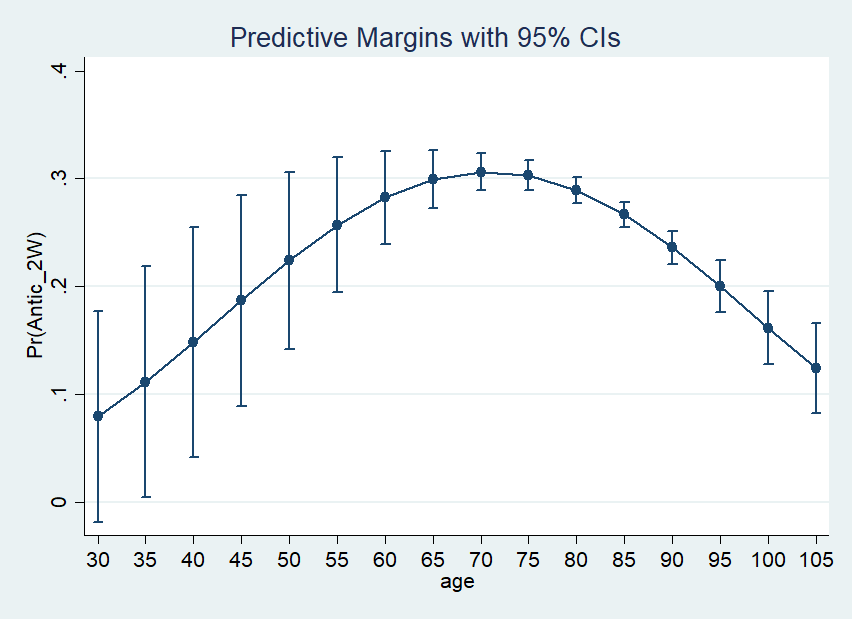


DM: diabetes mellitus; HbA1c: glycated hemoglobin; LDL: low-density lipoprotein; TSH: thyroid stimulating hormone; BZD: benzodiazepines; PPI: proton pump inhibitor; AMI: acute myocardial infarction; P2Y: clopidogrel, prasugrel or ticagrelor; NSAID: nonsteroidal anti-inflammatory drug; PAD: peripheral artery disease; Afib: atrial fibrillation.
